# Psychological stressors among faith-based health workers during Marburg virus outbreak in Ghana: A qualitative study

**DOI:** 10.1371/journal.pmen.0000042

**Published:** 2025-05-29

**Authors:** Herman Nuake Kofi Agboh, George Adjeisah Adjei, Grace Adjei Okai

**Affiliations:** Catholic Health Service Trust, Ghana, Accra-Ghana, West Africa; Makerere University, UGANDA

## Abstract

**Objectives:**

Psychological distress is a common occurrence among health workers during infectious disease outbreaks. Yet documented evidence on the scope of the challenge in faith-based healthcare organizations is scanty. Accordingly, this research used Lazarus and Folkman’s Transactional Model to assess faith-based health workers’ exposure to stressors during the outbreak of the Marburg Disease Virus in Ghana, the coping strategies adopted and psychological interventions employed to assist affected staff.

**Method:**

A phenomenological study, involving 15 clinical and nonclinical healthcare workers from the Christian Health Association of Ghana, was conducted. Interviews were arranged virtually, and data analyzed with Braun and Clarke’s (2006) thematic analysis.

**Findings:**

Participants revealed stress and mental health challenges during the Marburg disease outbreak, citing quarantine, fear of infection, and inadequate protective measures as stressors. Psychological impacts included insomnia, anxiety, and heightened health vigilance. Dissatisfaction arose from insufficient support and resources, such as isolation facilities, protective gear, and counselling. Work-related stress emerged from increased workload, staffing issues, and a lack of expertise. Concerns extended to family well-being and personal life.

**Conclusions:**

Enhancing the support infrastructure of faith-based health facilities in Ghana, including expanded psychological resources and the adoption of health emergency protocols would enable such facilities to secure health workers from mental distress during health emergencies.

## Introduction

Healthcare workers (HCWs) play leading roles in the fight against infectious diseases, making them one of the most at-risk populations during epidemics and pandemics[1,2]. According to Amnesty International [3], at least 230,000 HCWs were infected with COVID-19 and over 3,000 died from the disease. There is also a growing concern about the mental and psychological breakdown of HCWs, impacting their well-being, and culminating in burnouts, mental exhaustion, detachment from the job, and reduced professional efficacy during major disease outbreaks [4–6]. Evidence from recent health systems response to COVID-19 and Ebola, for instance, revealed burnout, post-traumatic stress disorders (PTSD), anxiety, depression, and decreased job satisfaction among HCWs in Low and middle-income countries (LMICs) [7,8]. In a narrative review on the “psychological impact, risk factors and coping strategies to COVID-19 pandemic on health workers in sub-Saharan Africa, Oyat, et al [7] analysed data from 5,323 research participants from Ethiopia, Uganda, Cameroon, Mali, and Togo, and found that between 16.3% and 71% of HCWs reported depressive symptoms, 21.9%-73.5% experienced anxiety disorders, 15.5%-63.7% experienced stress disorders, 12.4%-77% reported sleep disorders, and between 51.6% and 56.8% reported PTSD symptoms. Similarly, a study on the psychological effects of COVID-19 on HCWs and Community Care Workers in Wuhan using Beck Depression Inventory-II (BDI-II) and Beck Anxiety Inventory (BAI) shows that majority of frontline workers experienced fear of infections and infecting others, exhaustion from prolonged use of protective gears, and straining workloads, among others [9]. The study also shows prevalence of elevated depression (BDI-II scores≥14) and anxiety symptoms (BAI scores≥8) among HCWs, with over half (59.0%) of the respondents experiencing moderate to severe levels of perceived stress (PSS scores≥14) [9].

The cause of mental and psychological breakdowns of HCWs during epidemics and pandemics is multifaceted, stemming from high workloads, inadequate resources, and the pervasive fear of contagion, among others [10–12]. In their paper, “Preparedness and the importance of meeting the needs of healthcare workers: a qualitative study on Ebola”, Belfroid, et al. [13,14] interviewed 23 HCWs who had experienced different levels of stress and anxiety during the outbreak of Ebola disease and found that the healthcare facility did not have clear protocols to handle infectious disease outbreaks. Similarly, there were no guidelines on the requisite preparations to manage newly reported cases of Ebola adequately [14,15]. Amnesty International [3] investigated the preparedness of 63 countries to respond to COVID-19 pandemic and found a general shortage of PPEs and other materials, including gloves, goggles, face shields, gowns, and aprons for HCWs. These reports established direct associations between the emergency preparedness and response capacity of healthcare institutions and heightened levels of stress and anxiety among health staff.

Ghana’s health system infrastructure presents similar challenges to its preparedness and response capabilities, exposing health workers to extreme physical and psychological risks [1,2,16]. In addition to the lack of PPEs and other protective equipment, fear, stigma, and infection prevention and control protocols associated with infectious disease outbreaks, including social distancing, quarantine, and isolation measures, create emotional and psychological stressors, leading to anxiety, withdrawals, and depressive symptoms among HCWs during disease outbreaks [1,2,8,17–19]. According to Adom, et al [18], stigma, ranging from name-calling to refusing access to public spaces during the outbreak of COVID-19 was responsible for depression among HCWs [20]. Similarly, Arthur-Mensah Jnr, et al. [17] surveyed 38 HCWs in Ghana and observed that they all experienced fear. At least 60.5% experienced moderate to severe depression, 27% experienced high levels of state anxiety, 26.3% exhibited high levels of trait anxiety, and over 97% experienced symptoms of moderate to high-stress levels. In addition, in a qualitative study of 44 healthcare workers from five administrative regions of Ghana, Adongo, et al. [21] reported that 38 (86.4%) expressed fear and unwillingness to work in Ebola treatment centres.

Despite the teaming record of documented evidence linking weaknesses in health systems’ preparedness and response capacities to various levels of physical and psychological health outcomes among HCWs in Ghana [16,22,23], none of these studies were exclusively focused on faith-based healthcare institutions in Ghana. Given this knowledge gap, we used Lazarus and Folkman’s Transactional Model of Stress and Coping [24] to explore the stress experiences of healthcare workers (HCWs) during the outbreak of Marburg Virus Disease (MVD) in Ghana [24]. The framework conceptualizes stress as a function of the dynamic interplay between HCWs and their environments through cognitive appraisals and coping. This enabled the examination of perceived stressors during MVD outbreak and how the emergency response protocols of the Christian Health Association of Ghana (CHAG) influenced the mental health and well-being of its staff [24].

## Method and materials

To allow for the exploration of participants’ subjective realities, and knowing the kind of distresses experienced and coping mechanisms adopted during the outbreak, the phenomenological research design was employed [25,26]. We limited the scope of the study to the facility level.

Lazarus and Folkman’s Transactional Models of Stress and Coping guided data collection and analysis [24]. The model enabled the cognitive appraisal of participants’ responses to real and perceived dangers of MVD in two stages, namely the primary and secondary stages [24]. The primary stage appraised the extent to which MVD was considered dangerous or threatening to staff’s health and personal goals. The Secondary risk factors on the other hand included the primary stage’s threatening impulses, and the capacity of the facility, given its resources, to assist the staff to cope with the stressors.

### Participant selection and data collection

The study was conducted in the Christian Health Association of Ghana (CHAG). CHAG comprises 34 Church denominations, making it the second-largest healthcare collaborator with the Ghana Health Service. It employs 34,589 individuals across 374 primary, secondary, and tertiary healthcare facilities, and reaches a population of at least 11,308,640 Ghanaians [27]. The study is located in one of CHAG’s Primary Hospitals. The facility serves as the sole referral hospital in its administrative district. In 2022, it recorded 26,972 OPD attendances, with a bed capacity of 41, available bed days of 14,965, and 7,775 annual inpatient days.

The study targeted both clinical and nonclinical healthcare professionals directly engaged in managing MVD in Ghana. The purposive sampling technique was applied to select participants whose insights align with the research question. Authorization to access the facility was granted by CHAG, and participants were contacted through phone calls to obtain their consent to participate in the study. Fifteen participants were interviewed using a semi-structured guide with open-ended questions. The interview guide comprised four sections: participants’ demographic characteristics, emergency preparedness and response, and stressful outcomes, and mental health interventions. The total number of interviews was determined by data saturation [26].

The interviewees comprised HCWs in various roles, including the District Deputy Chief Disease Control Officer, District SNO Public Health, Hospital Administrator, Senior Health Service Administrator, Senior Medical Officer, Medical Director, Medical Doctor, Nurse Manager, Nursing Officers, Enrolled Nurse, Biomedical Scientist, and Human Resource Manager.

We conducted the interviews virtually on Zoom between April 20, 2023, and August 1, 2023. The interviews were recorded, transcribed, and coded. Each author verified the accuracy of the transcripts, and the data analysed using NVivo (version 14) [26]. To minimize external influences, participants were required to isolate themselves during interviews; if isolation was impractical, interviews were conducted after working hours. Participants provided written informed consent before participating in the research. An audit trail was carefully documented to capture the decisions made throughout the research.

### Reflexivity

As the interviewers were employees in the CHAG system, we acknowledge possible feelings of anxiety by participants. To address this, the interviewers took steps to maintain a neutral position and avoided questions that may heighten the discomfort of participants. Additionally, because the authors work within the CHAG system and understand the service delivery gaps in the organization, they made a conscious effort to focus data search and analyses solely on information obtained from participants, and evidence retrieved from published literature.

### Data analysis

This study utilized Braun and Clarke’s [28] thematic analysis for data thematization and analysis. Coding and data organization occurred at three levels. This was preceded by multiple independent reading of the 15 transcripts by each author to generate codes. Authors individually coded data, then cross-referenced and addressed differences in the results through comparison and discussion. Through the reconciliation, similar responses and implied meanings were grouped to form the main themes, and similar ideas within each it condensed into subthemes in line with Lazarus and Folkman’s Transactional Model of Stress and Coping [24]. This process was reviewed multiple times by the authors to prevent potential analytical biases.

### Ethics approval and consent to participate

Ethical approval was obtained from the Ghana Health Service Institutional Review Board (Review Number: GHS-ERC 007/02/23). Participants also granted written informed consent, and the necessary confidentiality measures, including substituting or omitting participants’ identifiers like facility names and residences to safeguard anonymity in the publication, applied.

### Findings

Table 1 outlines participant demographics. We interviewed a District Deputy Chief Disease Control Officer, a District SNO Public Health, a Hospital Administrator, a Senior Health Service Administrator, a Senior Medical Officer, a Medical Director, a Medical Doctor, a Nurse manager, 4 nursing officers, an enrolled nurse, one biomedical scientist, and a Human Resource Manager. Females (n = 9) outnumbered males (n = 6). The age distribution leaned towards 30–39 years (n = 9), with 40–49 years being the next prevalent group (n = 4). Marital status revealed 11 married participants, and 12 were parents at the time of the research.

**Table 1:** Participant List.

NAME	DESIGNATION	AGE (YEARS)	GENDER	MARITAL STATUS	PARENTAL STATUS	YEARS OF EMPLOYMENT
DDCO	District Deputy Chief Disease Control Officer	44	M	Married	Yes	2
DPHN	District SNO, Public Health	36	F	Married	Yes	5
HA	Hospital Administrator	43	M	Married	Yes	14
SHSA	Snr. Health Service Administrator	34	F	Married	Yes	5
SMO	Snr. Med. Officer	37	F	Married	Yes	4
MDir	Medical Director	42	M	Married	Yes	16
MD	Medical Doctor	46	M	Married	Yes	10
NM	Nurse Manager	37	F	Married	No	4
NO.1	Nurses Officer	29	F	Single	Yes	7
NO.2		36	F	Single	Yes	10
NO.3		34	F	Married	Yes	8
NO 4		39	F	Married	Yes	8
EN	Enrolled Nurse	26	F	Single	No	3
BS	Biomedical Scientist	31	M	Single	No	4
HRM	Human Resource	38	M	Married	Yes	5

Source: Field data, 2023

Table 2 highlights the main themes and subthemes from the analysis conducted. The themes were generated with the guidance of Lazarus and Folkman’s Transactional Model of Stress and Coping [24]. From the data, “stress outcomes and risk factors” and “counselling and psychological therapy” emerged as the primary and cognitive appraisal indicators [24], respectively.

**Table 2:** Emerging themes.

BASE THEME	SUB-THEME A	SUB-THEME B
Stressful Experiences	1.Stress outcomes and risk factors (Primary appraisal)	Workload and resource constraints Human resource capacity and staffing needs Fear and anxiety Impact on personal and family life Impacts on work output Communication and support from management
	2.Counselling and psychological therapy (Secondary appraisal and Coping)	Limited formal counselling services Informal peer support and communication Home quarantine and coping mechanisms

Source: Field data, 2023.

### Stress outcomes and risk factors

The narratives reflected a profound psychological toll on participants, emphasizing the need for comprehensive support, resources, and mental health interventions to address the complex interplay of stressors and mental health outcomes experienced during Marburg outbreak. The participants attributed the stress and mental health outcomes to many factors. Some of these include increased workload and resource constraints, staffing needs, fear of infection, and impact on family and work life, among others.

### Workload and Resource Constraints

According to the participants, work-related challenges such as increased workload, insufficient staffing, and a lack of expertise contributed to the overall stress. They mentioned that the facility lacked essential medical equipment to run tests, decontaminate the facility, and keep workers and patients safe. On the contribution of the facility’s resourcefulness to stress outcomes, a Nurse Manager (Head of Nurses) had this to say:

*“There should have been some compensation for us because of the workload. We have only one monitor and very few testing kits and PPEs. Because the unit itself is very small we were exposed to out-patients and relatives visiting us frequently and entering the wards at will…(MN)”.*

The professional nurses who worked directly with MVD patients expressed similar concerns about the inadequacy of protective equipment to shield workers and patients from infections. One of them had this to say:

*“We had everything but it wasn’t enough… a few overall suites and gowns, a small emergency unit, no isolation centre… Even when we talk about referring cases, sometimes getting an ambulance becomes very difficult…(NO 3).”*

The Medical Director of the facility also shared his experiences

*“We don’t have ventilators or oxygen concentrators so we still use the few cylinders, gloves from the COVID era and PPEs as much as possible (MDir)”.*

### Human resource capacity and staffing needs

While participants generally lauded the achievement of the few staff available, some called for special compensation for the work done. The majority expressed concerns about the capacity of the human resources of the facility, including the number of staff available and qualifications to effectively respond to the outbreak. On the staffing needs, a Medical Director and a Medical Doctor expressed their experiences as follows:

*“In terms of human resource capacity, the number is not enough at all…this place is a bit remote so when you post people here they don’t come. Most of our nurses are enrolled nurses (unskilled nurse assistants). We don’t have enough of the diploma and degree nurses, so as a doctor, if you are working with such people you need to go over and over to make sure things are being done right. As a referring centre, we don’t have the numbers, and we don’t have the expertise at all (MD)”.*

The Medical director shared detailed information on the human resource expertise lacking in the facility. He explained this as follows:

*“Altogether, there were 3 permanent MOs (Medical Officers) and 2 PAs (Physician Assistants) who are the prescribers. During the outbreak, one of the resident MOs was on leave, and the other 2 MOs and 2 PAs together with all the emergency staff were all exposed because they took care of infected patients. I was somehow exposed, but I didn’t self-isolate. We called CHAG and the regional office because basically, the whole OPD and emergency staff were in self-isolation. But eventually, we were able to get some locum staff. Also, we don’t have a resident disease control officer within the facility itself. We have a field technician who acts as a disease control officer so that was a challenge. Our public health nurse just completed school so as of that time we didn’t have a designated public health nurse or someone with a degree in public health. We didn’t have a designated public health nurse because we don’t actually have a standard public health unit. We had a CHN (Community Health Nurse) who played the role very well (MDir)”.*

The nurse manager also shared her experiences as follows

*“…We were 15 in number in the emergency and 7 of our staff were quarantined…(MN)”.*

### Fear and Anxiety

Psychological outcomes, such as insomnia, anxiety, and heightened vigilance regarding personal health, were expressed. Majority of the participants, especially frontline staff with less than two years of work experience and those who had direct contact with infected patients, expressed fear and anxiety. Some of them resorted to immune boosters. The nurse manager, for instance, who attended to the first MVD patient had this to say:

*“For me, I had a splash of blood on my eye and was quarantined for 14 days… Psychologically, you will think that you have the condition. At one time, I was talking to a colleague and he said that my eyes were reddish, so they were telling me that I had gotten the condition… For the first five days, I couldn’t sleep because you get this psychological symptom, at times you get this abdominal pain, you get severe headache and you will think that’s the symptom of the virus… when I go to the washroom I will be checking my urine and faeces for blood in it (NM)”.*

One of the nurse officers also complained about the psychological impacts of quarantine on her mental health. She narrated her experiences as follows:

*“Yes, it affected my life because I was a newly posted nurse for the first time. I was asked to be indoors for two weeks, movement as a person curtailed, and many important places I couldn’t visit, and nobody was allowed to come to visit us. It was only myself. It affected everything. Being told that the disease does not have medicine or cure …The fear was there…The anxiety was there… I was having insomnia as a result of fear…but you just have to psych yourself…So many of us stayed in the house for quarantine and the workload also increased (NO 1)”.*

We interviewed one Enrolled Nurse (EN) who shared similar sentiments (Enrolled nurses are unskilled support staff). Akin to other sentiments shared, one of the ENs explained how exposure to new and risky tasks with infected patients affected their mental wellness. Some of the enrolled nurses experienced headaches, fear, and trauma. One of the ENs narrated her ordeal as follows:

*“I have not seen a patient who has expired and is still bleeding, so that was what was scaring me… I don’t think we were protected enough…I had not been quarantined before, this was my first time, I wasn’t that ok but there was nothing I could do…I was really scared… I even called my father and my father gave me COA Mixture (Immune Booster)… I get a headache I would wonder if it was the Marburg… I watched movies and cooked to cope with the fear… with all of this development, exposure and everything, there was no counselling unit for traumatized staff (EN).”*

### Impact on personal and family life

Concerns about family well-being and personal life were raised. The quarantine affected communication with friends and family, with some of them, avoiding communication with the outside world to prevent panic. The Senior Medical Officer shared her experiences as follows:

*“When it happened, during the initial stages, we were all scared because we knew the mortality rate of Marburg disease, and it was a new thing altogether. Throughout my practice, I have never even managed a case like that, and I have never seen some before…so in the initial stages, we had some psychological trauma…my sleep was affected. We were also worried about family and friends in case something happened to us and you have people like parents who are dependent on us (SMO).”*

One of the Medical Officers shared similar sentiments

*“It affected my whole life…because my family would go through the same trauma and I wouldn’t have slept from excessive calls if I had told my mother, brothers and sisters, so I didn’t tell them. I only told my wife and I had to encourage her. If I didn’t tell her, probably she might want to come around that was why I told her…(MD)”.*

The nurse manager also shared the effects of the infection prevention and control measures on her personal and family life:

*“…my family and friends were afraid to come to me so we communicated on the phone, and they encouraged me (NM)”.*

### Impacts on work output

Participants expressed dissatisfaction with the measures instituted to prevent institutional and community spread of the disease. The absence of equipment like isolation facilities, protective equipment, and counselling services affected the staff’s sense of security and affected their work output. A senior nurse officer shared her experience:

*“It affected my work because I became very conscious when attending to patients. Because I didn’t know the patient I would attend to…after every procedure I washed my hands, wore gloves, nose mask everywhere I went…You will sleep and in the middle of the night you wake up and be thinking of all the signs and symptoms… when you urinate you check if there’s blood in it, and when you defecate you check if there’s blood before you flash… so it wasn’t easy (NO 2).”*

Similarly, one of the medical doctors explained how the experience compelled him to take infection prevention measures more seriously than in the pre-MVD era. He had this to say:

*“Psychologically, it affected my job because I took infection prevention measures very seriously… the facility doesn’t have gloves… so I will sanitize my hands and sanitize all my stethoscope…In the initial stages, I was scared…l was expecting to hear the results of the initial cases to know whether the outcome would be positive for the two patients…so during this period, sleeping was not good…I’m always wearing my mask…I was wearing masks, PPEs, and gloves when I saw patients (MD).”*

### Counselling and psychological therapy

The participants shared experiences and perceptions regarding mental health support systems in the facility, revealing a significant impact of the preparedness and response strategies of the CHAG facility on staff mental health. While some found solace in informal peer support networks, others highlighted the lack of formal counselling services as a major challenge. It was also observed that while the facility management encouraged self-monitoring, informal conversations with familiar individuals substituted formal counselling for some participants. However beneficial these interventions were, they were not enough, leading to a need for more structured mental health support. Other experiences include the effects of home quarantine on stress outcomes, access to psychological medication and the role played by the Chaplaincy unit.

### Limited formal counselling services

The facility has a mental health unit, but participants had mixed views on its accessibility and usefulness. As a result, the staff were encouraged to engage in self-monitoring. The overall evaluation highlighted a need for more structured and accessible mental health support within the facility. A medical officer and the Biostatistician had these to say:

*There was no counselling for affected staff. But I think we were told that we should keep talking to people that we know and people who were involved so that in case they need any assistance or they feel traumatized they can come around and talk to my boss, myself or the nurse manager. I think we called people who went through these traumatic experiences (MD).*

The facility’s Biomedical Scientist confirmed the lack of adequate counselling service for staff who went through stressful experiences during the outbreak. He explained this as follows:

*I can’t really confirm if we have a formal counselling programme for all that went into quarantine. The facility has a mental health unit where we can walk in and talk to the people there. I didn’t feel at the time I needed that but I was able to speak to the nurse manager on some issues I couldn’t speak to others about (BS).*

### Informal peer support and communication

There were mixed reports on the level of engagement between exposed staff and management of the facility. While some of them admitted receiving frequent calls from the facility’s management, others complained about the infrequent check-ins from the management team. A nurse officer expressed disappointment with the number of times the facility managers checked on her during quarantine. She narrated her experience as follows:

*“When we were quarantined, nobody called to even ask how we were feeling. They only came around two or three days into the quarantine. After we came to the house, they came to check on us and brought us a few foodstuffs. That was all till the two weeks were over. Honestly, I felt so bad about it, because I was expecting someone like the nurse manager or the in-charge to call and ask us how we were feeling or if we were experiencing anything (NO 3)”.*

Participants who felt supported found solace in talking to peers, and regularly checking on each other and sharing encouragement. A nursing officer and a medical doctor shared their experiences as follows:

*I was calling those who were involved, we were talking to ourselves, encouraging ourselves so every day we would call one or two times to check on each other and to know if anyone had the signs and symptoms (NO 2).*

*Aside from unannounced visits, there were regular calls to find out whether they were still around and monitoring individuals to find out how they were feeling and any signs they were experiencing at any moment… (MD).*

Aside from informal support systems, there was a Chaplain in the facility to deliver counselling services to staff. The Human Resource Manager had this to say:

*“We have the Chaplin around who was available every day to counsel the staff. Things that are beyond educators, they direct them to the Chaplin to take over (HR).”*

### Home quarantine and coping mechanisms

Some participants resorted to watching movies to alleviate stress but found it to be a temporary escape. A nurse officer and a medical doctor shared their experiences as follows:

*Watching movies and reading. That was the only thing I was doing…However, a few friends that I was able to open up to call to check up on me and encourage me…the management only told us that when we start experiencing the symptoms, we should call them, but I overlooked certain symptoms because there could be many conditions (NO 1).*

*I watched movies like cartoons and others just to release stress but when you finish watching them, it comes back to you… (MD).*

Overall, the accounts reflect a mix of experiences regarding mental health support, ranging from informal peer support networks to a perceived lack of formal counselling services. While some found comfort in personal coping strategies and informal communication, there was a recognized need for more structured and accessible mental health support within the facility.

## Discussion

### Primary Appraisal - Stress outcomes and risk factors

Findings from the primary appraisal revealed stressful experiences among healthcare workers (HCWs) during the Marburg virus disease (MVD) outbreak in Ghana [24]. Stressors such as quarantine, fear of infection, and inadequate protective measures were observed. For instance, the fear of contagion, compounded by the lack of adequate resources and support [29], was observed to have intensified stress levels, leading to symptoms of depression, anxiety, and sleep disturbances [4–6]. The pervasiveness of anxiety, insomnia, and hyper-vigilance among the HCWs is likely a reflection of broader health system lapses in the facility, similar to healthcare facilities in low and middle-income countries [1,3].

In addition, similar to findings from studies on COVID-19 and Ebola [1,7,16,17] where participants expressed feelings of fear, stress, and anxiety from lack of proper protective measures and support systems, this research also revealed a strong relationship between the lack of protective materials and stress symptoms among HCWs. Our findings revealed that the health workers grappled with the uncertainties surrounding MVD and its implications for health and wellbeing in the absence of clear protocols. Accordingly, their vigilance was heightened, leading to the constant monitoring of symptoms and pervasive anxiety, disrupted sleep patterns and exacerbated feelings of helplessness and isolation. These findings mirror the psychological impact of infectious disease outbreaks documented in previous research, highlighting the adverse mental health outcomes experienced by HCWs when preparedness and response strategies are poor [2,10–12].

Moreover, the Marburg outbreak heightened existing health human resource challenges within the target facility, including increased workloads and staffing shortages. This finding illustrates systemic deficiencies in the emergency preparedness and response capacity of CHAG. For instance, parallel to the study of Ebola outbreaks in Ghana by Nyarko, et al [22], inadequate staffing levels and reliance on unskilled healthcare workers during the outbreak exacerbated stress levels among HCWs, creating concerns about patient care and personal safety during disease outbreaks [16,21,22]. Beyond this, the lack of clear protocols, inadequate resources, limited access to protective equipment like isolation facilities, shortages of personal protective equipment (PPE), and challenges in accessing essential supplies also resonated with reports of previous studies on health systems’ vulnerabilities during pandemics [1,17]. For instance, we observed that the absence of clear protocols and structured support systems [11–13], including counselling services and psychological interventions, intensified the psychological burden of the HCWs, and hindered their ability to cope effectively with stress [3,13,14].

Addressing the challenges from the primary appraisal requires reforms at the policy and institutional levels [24]. A secondary appraisal focusing on strengthening the capacity of faith-based healthcare institutions’ resilience will address the risk factors of stress during health emergencies, the psychological toll of stress on HCWs and workers’ resilience against psychological breakdowns [3,22,23].

### Secondary Appraisal and Coping - Counselling and psychological therapy

Given the number of mental stressors observed from the primary appraisal, a secondary appraisal with a focus on psychotherapy and social capital was done to determine the capacity of the facility to assist affected staff [24]. Participants’ accounts revealed a lack of structured counselling and psychological support mechanisms during the Marburg virus disease outbreak in the Christian Health Association of Ghana (CHAG) facility. While informal peer support emerged as a coping strategy, the absence of formal counselling pathways left many unsupported and overlooked. Similarly, although the participants mentioned the provision for home quarantine, it lacked proper psychological medication, offering only basic provisions like vitamin C and food, and failing to address the emotional and psychological needs of affected staff.

The lack of access to counselling and psychological therapy among participants reflects broader structural deficiencies within CHAG’s mental health support infrastructure. While some HCWs mentioned the availability of a mental health unit and Chaplain for counselling, others expressed limited access to these programmes. It is also worth mentioning that the availability of a mental health unit and Chaplain services may demonstrate recognition of the importance of psychological support within the facility [3,18,19]. However, the participants’ mixed views on accessibility and usefulness highlight potential barriers to these interventions during health emergencies. Evidence from previous studies [3,21] show that discrepancies between perceived and actual accessibility is a reflection of systemic challenges in integrating mental health services into routine healthcare practices, further echoing broader issues in healthcare infrastructure and resource allocation in the research facility [3,21]

Due to the absence of a structured counselling system, majority of the HCWs relied on informal support systems. Informal peer support and self-monitoring, though beneficial, may not adequately address the complex psychological needs arising from infectious disease outbreaks [13]. Such gaps in psychological support mechanisms often lead to heightened stress, burnout, and reduced job satisfaction among HCWs, ultimately affecting health facilities’ resilience and capacity to respond effectively to health emergencies [7]. Thus, while participants’ reliance on informal coping mechanisms reflects resilience, it is not a sustainable approach to managing stress among HCWs during major health emergencies.

Overall, experiences shared by the research participants revealed critical areas for improvement within CHAG’s preparedness and response to mental health concerns during health emergencies. Firstly, the lack of clear protocols for managing mental health crises during health emergencies exposes systemic vulnerabilities in emergency preparedness and response strategies. In addition, consistent with findings from the literature, deficiencies in resource availability, including personal protective equipment (PPE) and psychological support services, contributed to heightened levels of stress and anxiety among the HCWs [3,14,22]. Addressing these gaps requires multi-faceted strategies comprising enhanced resource allocation, capacity building in mental health literacy, and the integration of psychological support services into emergency response frameworks, among others [1,18,19]. Prioritizing mental health support services, including counselling and psychosocial interventions [3,22,23], fostering a culture of psychological resilience, and peer support, can empower HCWs to cope effectively with the stressors inherent in their roles and promote a supportive work environment for optimal patient care delivery. At the policy and institutional level, strengthening healthcare systems’ resilience through improved emergency preparedness and resource allocation is paramount to mitigate stressors and enhance workforce capacity [22]. Investments in PPE provision, isolation facilities, and training programs are essential to safeguard HCWs’ well-being and ensure effective response to future outbreaks [8,17].

### Limitations

The study has inherent limitations. Interviews were conducted one year after the MVD outbreak in Ghana, potentially leading to recall bias and forgetfulness. To address this, a concise background summary preceded substantive questions for each interviewee. Then again, our study’s scope was confined to one health service agency, limiting generalizability. To address this, future research should target cross-national examinations of emergency response capacities in health systems across Africa and other LMICs.

## Conclusion

The experiences of healthcare workers (HCWs) in this research demonstrate a complex interplay of mental health stressors and health systems challenges during the outbreak of Marburg virus disease in Ghana.

From Lazarus and Folkman’s Transactional Model of Stress and Coping [24], we observed that strengthening the Christian Health Association of Ghana’s mental health support infrastructure may require the establishment of formal counselling programmes tailored to the unique needs of HCWs during infectious disease outbreaks. These programmes should provide accessible and culturally sensitive psychological interventions, including cognitive-behavioural therapy and mindfulness-based stress reduction techniques. In addition, proactive measures such as regular mental health screenings and awareness campaigns can help destigmatize mental health issues and encourage early intervention. Finally, investing in training and capacity-building initiatives for healthcare personnel on psychological first aid and trauma-informed care can enhance the ability of HCWs to support colleagues and patients during crises. By prioritizing mental health and well-being, the facility can foster a resilient workforce that is better equipped to navigate the challenges of infectious disease outbreaks in Ghana.

## Supporting information

S1 Data De-identified data set. (DOCX)
