# Supplementary material for: Psychological stressors among faith-based health workers during Marburg virus outbreak in Ghana: A qualitative study
Source: PLOS Ment Health. 2025 May 29;2(5):e0000042. doi: 10.1371/journal.pmen.0000042 (PMC12798296; doi:10.1371/journal.pmen.0000042)
Supplement: S1 Data — (DOCX) [file pmen.0000042.s001.docx]

**TRANSCRIPT**

Preparedness and response to Marburg disease in Ghana: Qualitative study of psychological stressors among faith-based healthcare workers [PMEN-D-24-00065]

NURSE MANAGERS (NM)

|  | |
| --- | --- |
| RESPONDENTS | R |
| INTERVIEWERS | I |

*Demographics* *were deleted to protect the privacy of the respondents and the facility*

R: The first patient’s name is ......... He was admitted on the 26th of July, 2022. He was a 26-year-old young man. I came in to work on Monday, and that’s when I took over his case. My colleagues informed me that he had been experiencing severe symptoms, including diarrhea, vomiting, and coughing, which had been ongoing for about three days. That same day, the prescriber came to review him, and the patient reported that he had started vomiting blood. He also complained of severe lower abdominal pain and was passing bloody stool. We noticed that he had jaundice because both of his eyes were visibly yellow. So, that’s what we were initially managing, and the diagnosis made was acute gastroenteritis.

The patient also had a distended abdomen, so a second diagnosis of paraumbilical hernia was considered. We were monitoring him after rounds, and shortly afterward, we observed that he had a seizure. After the seizure, he became unresponsive. As we continued managing him, we noticed he started bleeding from his mouth—there was red and white blood. I immediately called one of my colleagues, to assist me with the assessment. When we examined him, we saw that his tongue had been bitten, likely during the seizure. We began treating the injured area on his tongue, but unfortunately, each time he bit down, the bleeding resumed, and we couldn’t effectively control it. We decided to pass a nasogastric tube (NGT) and drained approximately 1500 ml of a mixture of red and white blood from his gastrointestinal tract. We also administered blood and gave him 10 ml of vitamin K to help with clotting.

I: Thank you for the background on the patient’s condition. Now, we are focusing on the administrative procedures that led to the injection. Could you walk us through the medical tests or protocols you undertook after suspecting something abnormal in the patient's symptoms?

R: It was around 2:30 in the afternoon when we noticed that he had bled into his eyes, which was quite unusual. I immediately contacted the prescriber and updated him on the situation. He advised us to reach out to the district director, which we did through our medical director. Unfortunately, the patient passed away later. The district director informed us that a sample would be collected the following day to investigate further, as there was a suspicion of Ebola. We were told that a team would come to collect the sample for testing. About a week later, we received confirmation from the lab that it was, in fact, Marburg Virus.

R: Currently, we don’t have a specific isolation unit in our facility. The emergency unit consists of two rooms. One room has three beds, and the other room has three beds as well. Given the circumstances, we decided to reserve one room as a temporary isolation unit to handle this case. We prepared it specifically for that purpose, so it was set aside solely for isolation. All staff members who were involved in the care of the patient were quarantined for a period of two weeks, which is 14 days.

I: Were you personally involved in handling the case?

R: Yes, I was directly involved. At one point, I had a splash of the patient’s blood in my eye.

G: Were you quarantined at the facility or were you asked to quarantine at home?

R: I was quarantined at home for the duration.

I: Aside from converting one of the rooms in the emergency unit into an isolation room, were there any additional measures put in place?

R: Yes, there were. The district director supplied us with personal protective equipment (PPE). We were instructed to use it if we encountered any patient with symptoms indicating upper gastrointestinal bleeding. In such cases, we would isolate the patient in that room and send a sample to [Noguchi] for analysis. If the test came back positive, we would then know how to proceed in handling the case. Additionally, they provided us with coveralls and gave us proper training on how to put them on and remove them safely. So, we had quite a few precautionary measures in place, and they were strictly enforced.

I: Was the equipment supplied by the district or was it provided by your facility?

R: The equipment was supplied by the district. Specifically, it was Mr. ......., the district director, who made these resources available to us.

I: Did these new infection control measures affect you in any way?

R: No, they didn’t affect me personally.

I: Do you think it was easier to adapt to these measures because of your prior experience with the pandemic?

R: Yes, I do think so. After the case was detected, the management held a meeting with us to discuss containment strategies. We were cautious about managing the information to prevent any public anxiety. It was handled carefully to ensure it didn’t spread, and as a result, it remained controlled.

I: Were you also quarantined?

R: Yes, I was quarantined for the full 14 days.

I: Did you have to separate from family, especially considering you were newly married?

R: No, actually, the case was detected in July, and I didn’t get married until September, so there was no need for separation from any family member at that time.

I: How did you handle being isolated from friends? Were you able to stay in touch with them?

R: Yes, we kept in touch over the phone. My friends were hesitant to visit because of the situation, but they encouraged me from a distance. They kept my spirits up throughout the quarantine period.

I: Being unable to participate in your usual interactions with co-workers, patients, and other daily activities—did this situation affect you in any particular way?

R: Yes, it did affect me in various ways, especially psychologically. But I was able to manage and get through it.

I: Could you share some specific ways in which it affected you?

R: Psychologically, it was quite challenging. I kept thinking that I might have the infection. Some of my colleagues even told me that my eyes looked a bit yellow, which made me worry even more that I might have contracted the virus.

I: I can imagine that must have been frightening for you.

R: Yes, it was, especially since for me, I had some of the patient’s blood splash into my eye. I was genuinely concerned that I might have contracted the virus.

I: Were you able to sleep well during the quarantine period?

R: Not really. For the first five days, I had trouble sleeping because I was constantly worried. I started experiencing symptoms like abdominal pain and headaches, and I thought they could be signs of the virus.

I: So, during that time, whenever you noticed something slightly unusual in your body, you immediately linked it to the possibility of infection?

R: Yes, exactly.

I: Did this heightened awareness about your health make you rethink any future plans or decisions?

R: Yes, it did. I became extremely conscious of any change in my body. I even started checking my urine and stool regularly to look for any signs of blood, just in case.

I: When you resumed work after the quarantine period, did you feel hesitant or fearful when handling patients who displayed similar symptoms?

R: No, not really. After the 14-day quarantine, I came back with a renewed mindset. I felt that if I hadn’t developed any symptoms despite the exposure, then God had protected me. So, I returned fully prepared to work and continue treating patients. However, I was definitely more cautious and protective than I used to be.

I: Did the facility provide any support services to you after your exposure to the virus?

R: Yes. While I was at home, they provided some provisions to support me. They brought things like rice, oil, Milo, milk, and vegetables. It was a supportive gesture from the facility, and it was quite helpful.

I: Did your family know about your exposure?

R: No, I chose not to inform any of my family members because I didn’t want to worry them unnecessarily.

I: Why were you concerned about informing them?

R: Yes, exactly. I was worried about how it would affect them, especially my mother and uncle. They would have definitely come to check on me, which would have affected them psychologically. So, I decided to keep them out of it.

I: How about your friends? Were they able to stay supportive?

R: Yes, my friends were supportive. We communicated over the phone, and they encouraged me, saying that since I hadn’t caught COVID during that outbreak, I shouldn’t be afraid of this one.

I: During that period, were there any support services you expected to receive from your friends, family, or institution that were not provided?

R: Yes, I had certain expectations. If not for myself, then at least for the facility where I work. We don’t have an isolation unit, and I expected that the ministry might provide us with one. When we encounter such cases, having a designated isolation unit would enable us to care for patients in a controlled and secure environment. Additionally, some form of compensation for those of us who were directly involved in handling the case would have been appreciated. Given that the case was detected here in our facility, There should have been some compensation for us because of the workload as acknowledgment for the risks we took and the work we did.

I: During that period, while you were working at the facility, were there any institutional policies in place on how to handle or manage such cases? Specifically, were there any policies concerning the management of the Marburg outbreak that you felt uncomfortable with or that you found challenging?

R: No, I didn’t feel uncomfortable with any of the existing policies. Everything was manageable from that perspective.

I: Thank you. Now, when you look at the hospital setting—the physical infrastructure, non-physical infrastructure, and programs—in what ways do the environment, the equipment, the architecture, or the resources make the facility more exposed to rapid outbreaks?

R: Our facility is quite congested. Everything operates in one block. We have the Outpatient Department (OPD), the emergency unit, the lab, and the consulting rooms all situated on the same block. Even the room we set aside for an isolation unit is in this same block. This arrangement is really not ideal at all, especially when we are trying to prevent the spread of infections. With this setup, the risk of spreading an infectious disease is significantly higher.

I: Is your facility a referral unit?

R: Yes, it is a referral center for the entire ............ area.

I: Do you feel that this designation exposes the facility to additional risks? Could you elaborate on that based on your professional judgment?

R: Yes, absolutely. Since every serious case from the district is referred here, it naturally increases our exposure to various diseases and infections. We receive a higher volume of cases from multiple locations, which means a greater chance of encountering infectious cases.

I: Could you give an example of such instances?

R: For example, during the COVID-19 era, many facilities around .......... referred cases with upper respiratory tract infections to us. Some of those cases eventually tested positive for COVID. So, having them referred here did increase the risk of exposure.

I: Were those patients quarantined when they arrived during the COVID outbreak?

R: Yes, we used the small isolation room that I mentioned earlier. That’s where they were kept.

I: Are you aware of whether your facility has an emergency disaster management and response plan or document to follow in cases of outbreaks or other emergencies?

R: No, I’m not aware of any specific document or formal response plan for emergency disaster management in our facility.

I: Were you working at this facility during the COVID-19 outbreak? And if so, did that experience give you the impression that certain protocols were being consistently followed during such situations?

R: Yes, I was here during the COVID outbreak. We did have certain protocols in place at that time. For instance, we had a designated triaging area where we assessed every patient coming into the facility. If a patient displayed symptoms suggestive of COVID-19, we would bring them to the emergency unit and then transfer them to the isolation room. We would then collect a sample for testing, and the rest of the procedures would follow accordingly.

I: So, do you use the same protocol for all disease outbreaks, or do you develop new protocols based on each specific outbreak?

R: We tend to develop protocols as each outbreak arises. In other words, it’s more of a reactive approach. When there isn’t an active outbreak, we don’t have a specific protocol in place. But when an outbreak is identified, we take action and strengthen our protocols according to the situation.

I: So, it sounds like you implement protocols only when there’s an immediate need. Would having a standard protocol in place improve preparedness?

R: Yes, we do have standard procedures as part of our normal nursing care, such as general triaging for all patients. This helps us assess if patients exhibit symptoms of any communicable diseases. When an outbreak like COVID or Marburg occurs, we strengthen these procedures significantly. But once the outbreak subsides, we continue triaging, although not as rigorously as we do during an active outbreak.

I: Apart from the procedures you described during the Marburg outbreak, how was information shared? Which department or personnel were involved in this process?

R: Initially, we held a meeting with all the Heads of Departments (HODs) to discuss the situation. We printed out information on the Marburg outbreak and posted it on the facility’s walls. We also began educating patients who were coming into the facility, advising them to remain calm and take the necessary precautions.

I: Apart from advising patients to remain calm and observe quarantine, were there any other security measures implemented to ensure the safety of both staff and patients?

R: Yes. For example, when patients arrived by taxi, we would go out to meet the driver and educate them on infection prevention. We would then use a chlorine solution to disinfect the vehicle.

I: Your department is the emergency unit, correct?

R: Yes, that’s correct.

I: Were you given any specific assignments following the outbreak?

R: Specific assignments?

I: Yes, since you had that departmental meeting to discuss managing the outbreak, was your department given specific roles in handling it?

R: Yes, we were given specific duties. One room in the emergency unit was turned into an isolation room. We were provided with PPE, including coveralls, to wear when treating patients suspected of having Marburg. We also received training on how to care for such patients safely and effectively.

I: So, you received training as well?

R: Yes, we were provided with training on handling cases and managing the isolation protocols.

I: Did you feel that you had enough human resources to handle the situation effectively?

R: No, we didn’t have enough human resources.

I: Could you elaborate on that?

R: This situation highlighted an ongoing issue with our facility’s resources. For instance, during COVID, we received only one set of PPE, such as coveralls, when there was an active case. The same happened with the Marburg case. PPE is only provided to us when there’s an urgent need, instead of maintaining a regular stock in case of emergencies. This kind of approach leaves us unprepared.

I: Regarding staffing, did you have enough personnel and the right mix of professionals to handle the outbreak?

R: No, not entirely. We had about 15 staff members in the emergency unit, but seven of us had to be quarantined. Our nurse manager had to go to other departments and bring in additional staff to help keep the emergency unit running smoothly.

I: At the time of the Marburg detection, had you interacted with staff from other areas?

R: Yes, I had interacted with staff from other areas as part of my work.

I: So, apart from the seven emergency staff members who were quarantined, were there others who had also interacted with the patient who were required to undergo quarantine?

R: Only one staff member from the OPD had to be quarantined. This person was involved with the second patient and had escorted them to the emergency unit. However, the other staff members didn’t have close contact with the patient, so there was no need to quarantine them.

I: You mentioned the procedure for collecting and sending samples. How was the process with the District Health Service (DHS)? Did it go smoothly, or were there challenges in getting the samples sent out and receiving feedback?

R: The process was smooth. I remember that on the first day we contacted the DHS, they informed us they would collect the sample the following day. True to their word, they arrived as scheduled, and everything went according to plan. We didn’t face any issues with sample collection or sending it to Noguchi for further testing.

I: Did you encounter any challenges with decontamination, evacuation, or safety protocols during the Marburg outbreak? You mentioned that the emergency ward was partially converted into an ICU for quarantine purposes. Were there other challenges related to evacuation, decontamination, or safety?

R: No, we didn’t face any significant challenges. I recall that a team from Obuasi came to assist us with the decontamination of the entire facility, so that went smoothly as well.

I: It seems like your facility has built a solid relationship with the government, particularly with the DHS. Could you share any insights on why you’ve received such consistent support?

R: I can’t say for certain why we’ve been able to get this level of support, but I know that our district director has maintained a good relationship with us, which might contribute to it. That strong connection likely helped us get the resources we needed.

I: I understand. You mentioned the lack of certain resources, particularly for the ICU and the emergency unit when it comes to quarantining patients. Aside from these, what other devices or equipment does your department or facility lack that are necessary to prevent, detect, and respond effectively to outbreaks like Marburg?

R: Currently, in the emergency unit, we have only one monitor that serves all the patients. We also need an X-ray machine, which would be very useful in detecting cases involving respiratory tract infections. But most importantly, we need a proper isolation unit. Having an adequate and dedicated isolation unit would make a huge difference in how we manage infectious cases.

I: So, the isolation area you were able to set up, even though it is within a stable facility—could we consider it a makeshift arrangement? From your professional judgment, do you think this setup is secure enough for isolation purposes?

R: No, it’s not secure enough.

I: Could you explain why?

R: The unit itself is very small, which already makes it less ideal for managing infectious cases. We are also exposed to outpatients and relatives visiting frequently; they come into the ward without much restriction. This kind of environment is not conducive for proper isolation because it increases the risk of spreading infections to others in the facility.

I: How effective was your laboratory in handling testing and other tasks during the Marburg disease outbreak?

R: For cases like Marburg, our lab isn't able to detect the virus directly. What we do is store the samples in our fridge, and when necessary, they are taken to Accra for testing.

I: I see. And was that arrangement effective? Did you encounter any challenges with it?

R: No, it worked smoothly. We had no challenges with this process.

I: What about your ambulance system? How helpful was it in both detecting and managing the outbreak?

R: We usually call the national ambulance service whenever there’s a referral to another facility. They respond when we call, but there’s sometimes a delay. They typically want to confirm with the receiving facility to ensure they’re prepared to accept the case. This confirmation process can delay our referrals to other hospitals.

I: Did you have enough testing kits available during this period? For instance, during the COVID-19 period, did your facility have access to an adequate supply of testing kits?

R: No, we didn’t have enough testing kits. Even the kits we did receive arrived late—they didn’t come at the right time. We had to wait, which was challenging.

I: I assume we could extend that to personal protective equipment (PPE) as well?

R: Yes, exactly. As I mentioned earlier, we only receive PPE like coveralls when there’s an outbreak. They are not readily available on a continuous basis.

I: Let’s go back to the quarantine period. Could you share some of the coping mechanisms you used to remain mentally stable, to continue functioning despite fear, to manage your sleep, and to handle the isolation from family, your patients, and work? How did you keep yourself going?

R: First and foremost, I put my trust in God, believing that He would save me from this situation. Secondly, I made sure to eat a lot of fruits to stay healthy. I was also very observant of any signs or symptoms, and thankfully, I didn’t develop any. My friends were very supportive—they encouraged me, telling me that nothing would happen to me. My nurse manager was also a great source of support, reminding me that even during the COVID-19 pandemic, I didn’t contract the virus, so I shouldn’t worry too much this time either.

I: How does your facility collaborate with other agencies, given your understanding of the operations and the local context? Is there anything about this collaboration that could be improved? Firstly, are you aware of any policy guidelines between GHS and CHAG facilities on handling outbreaks like this? What kind of relationship does CHAG have with the directorate or GHS regarding outbreak management?

R: No, I’m not aware of any specific policy guidelines between GHS and CHAG facilities for handling outbreaks like this.

I: From your professional experience, when there’s an outbreak like this, what kind of relationship do you think should exist between a CHAG facility and a GHS facility to ensure effective disease surveillance, detection, and response? What kind of relationship should these agencies have?

R: I believe that CHAG facilities should consult closely with GHS facilities, particularly when it comes to disease surveillance activities such as contact tracing. Collaboration is essential for effective management and tracking of cases.

I: Aside from the Marburg outbreak, although that is our main focus, I’m also interested in other outbreaks you have encountered, such as COVID-19. Based on your experience with these various outbreaks, have you observed any challenges in the collaborative work between your facility, which is a CHAG facility, and the directorate?

R: No, I haven’t observed any challenges. Our collaborations have been smooth, and I haven’t encountered any issues.

I: That’s good to hear. We’ve reached the end of our questions, but we’d like to offer you the chance to ask for clarification on anything we discussed. If there’s any question you need more understanding on, or if you’re curious about the reasoning behind certain questions, please feel free to ask.

R: Yes, actually, I do have a question. Is there anything that the government or the ....... plans to do for those of us who were quarantined or involved directly in this case?

I: That’s a great question. We’ll make a note of this and pass it on to the director. The director will provide us with a response, and we’ll make sure to get that information back to you.

R: Also, regarding the equipment you asked about—equipment that we need for the detection and management of outbreaks—will those be provided?

I: We’ll ask about that as well. Just to confirm, you’re asking about equipment for detecting and managing outbreaks, right?

R: Yes, that’s correct.

I: Thank you for clarifying. I’ll make sure to ask about it and then relay the response to you, either directly or through someone at your facility. Thank you so much for raising these points.

I: Thank you very much for your time. This has been a truly insightful discussion, and you’ve provided us with many specific details that will be invaluable in our analysis. I’ll hand over to my colleague now.

I: ............. *(name deleted)*, we’re truly grateful for your time and for sharing so openly with us.

R: Thank you, you’re welcome.

I: Could you kindly remind ............ that she is scheduled to join us at 11:30?

R: Certainly, I’ll remind her.

NURSES OFFICERS (NO 1)

|  | |
| --- | --- |
| RESPONDENTS | R |
| INTERVIEWERS | I |

I: Great, to start, can you tell us a bit more about yourself—your age, marital status…

R: Okay, I’m ready.

I: Thank you. And are you a parent? Also, could you tell us about your profession, your current designation or grade in your facility, and the number of years you’ve worked with the CHAG network? Just a little background to get us going. Can you still hear me clearly?

R: Yes, I can hear you.

I: Perfect. So, back to what I mentioned—just a bit about your age, profession, designation, and your work history with the CHAG network.

R: ..............*deleted to protect the identity of the participant*

I: Okay, thank you. And what role are you in at the facility?

R: I’m a nurse, specifically a registered nurse.

I: Alright, thank you for sharing that. Now, before we dive in, we want to clarify that this interview will focus on the detection and management of Marburg cases in your facility. Some of the questions we ask may touch on topics that could be emotionally sensitive or bring up uncomfortable memories. So, if at any point you feel uneasy or overwhelmed, please let us know, and we’ll pause or seek further assistance if needed.

R: Okay, thank you for letting me know.

I: Alright. Now, could you walk us through how Marburg was first detected in your facility, but without going into too much clinical detail? I’d like to understand the administrative side—what steps were taken as signs appeared, how samples were submitted, and the decisions around testing. Could you give us a bit of an overview of the administrative process that led to Marburg being detected?

R: Okay, let me start from the beginning. So, we had a case in the hospital. You know, as nurses, we work every day, seeing patients come in and go out. It was a typical situation where a patient was rushed to the emergency unit, and I happened to be on duty at that time. We started working on the patient, treating the symptoms based on what we saw. But then, the next day, we noticed something unusual—it didn’t match with the initial diagnosis we had for that patient.

I: I see.

R: Yes, and unfortunately, that patient passed away. But not long after, we had another patient come in with similar symptoms. This was when the ward in-charge raised an alarm. We had two similar cases, one of which had ended fatally, and this new one looked to be heading in the same direction. This was concerning, so the management decided to collect samples. They sent them for testing, and that’s when it was detected.

I: Thank you for explaining that. So, when the virus was detected in your facility, did it affect your personal lifestyle in any way? Or did it cause any changes in how you approached your work as a professional?

R: Yes, it definitely affected me. You see, I’m a newly posted nurse, and this was my first time working in this facility. So, it was quite unsettling. Imagine getting that kind of news shortly after you’ve just started settling in, and then suddenly, you’re receiving a call saying that you need to come in, and it’s not for work—it’s to discuss a possible virus exposure. That news was quite jarring. They told me and some of my colleagues to stay indoors for two weeks, to quarantine and monitor our health. So, there were restrictions on my movements, even in my personal life. I couldn’t go to certain places, and my routine was completely disrupted.

I: I can understand that must have been difficult.

R: Yes, it was. And to add to it, there was a shortage of staff at the facility as well because so many of us had to be quarantined. This increased the workload for those who remained.

I: Let’s also discuss how this affected your relationship with your colleagues. Were you quarantined at home or at the facility?

R: I was quarantined at home.

I: I see. And how did your family, parents, and friends react to this? Were they aware?

R: Well, I decided to keep it to myself. I didn’t tell my family initially because I knew my parents, especially, might react with fear or worry. So, no one from my family knew that I was in quarantine.

I: What about your colleagues or friends? Did anyone try to visit you?

R: No, no one was allowed to visit. I was instructed to stay indoors and not go outside, and I think my colleagues were probably informed as well, so they didn’t come by. I was completely alone, really.

I: That must have been hard. Were you okay with that situation?

R: Honestly, no, I wasn’t happy with it at all. Being indoors for two whole weeks without seeing anyone or talking to anyone face-to-face—it was tough. It’s hard to feel okay in that situation because I’m human, and isolation can get to you.

I: I completely understand. When you were in quarantine, did you feel afraid?

R: Yes, there was a lot of fear. Psychologically, it was challenging because, as a nurse, I’m usually the one giving reassurance to patients. But in that situation, I had to act as both a patient and a nurse to myself, constantly psyching myself up. The anxiety was definitely there.

I: Did this anxiety affect your sleep schedule?

R: Yes, absolutely.

I: So, were you able to sleep, or did you experience insomnia?

R: I had insomnia.

I: Was this due to fear?

R: Yes, it was the fear and anxiety. The whole situation was really unsettling, and that made it hard to relax and sleep properly.

I: I can imagine. When you’re told there’s no specific cure for the disease and you’re not sure whether you have it, being asked to go home for two weeks of isolation must have been a lot to handle, especially when you’re alone. It sounds incredibly uncomfortable.

R: Yes, it really was.

I: So, how did you cope with this whole process of being at home for two weeks? Apart from reading about the disease, was there anything you did to keep yourself positive or psychologically stable during that time?

R: Well, I tried to distract myself as much as possible. I watched movies and did some reading—those were the only things I could do to take my mind off the situation.

I: Did you receive any kind of support from family members who might have known, or from friends who were aware of your situation? Was there any kind of support, whether tangible or intangible?

R: Yes, I did get some support.

I: What kind of support did they provide?

R: As I mentioned earlier, my parents didn’t know about it. But I did open up to a few friends, and they would call me frequently to check up on me and offer words of encouragement. That meant a lot.

I: Reflecting on the decision to have you quarantine at home, and considering that you were working in the hospital and had observed how severe the virus could be, were you satisfied with the decision to quarantine at home?

R: Not really, but there was no other option available. We were just following the instructions given by the administration, and they assured us that if we began experiencing any symptoms, we should contact them for help.

I: Over that period, did you notice any symptoms yourself?

R: No, I didn’t experience any symptoms.

I: So it wasn’t like every little headache or discomfort made you think, "Is this Marburg?"

R: [Laughs] That’s actually a natural reaction.

I: [Laughs] Exactly, that’s what I’m talking about. Suddenly, every little thing makes you wonder if it could be Marburg.

R: That’s true, but I tried to psych myself up to ignore minor symptoms.

I: Yes, that makes sense.

R: Sometimes, you have to overlook certain things because they could be symptoms of many other conditions.

I: Can you still hear me clearly?

R: Yes, I can hear you.

I: Alright. Looking at your workplace environment—the architectural layout, how close or spaced the areas are, bed capacity, and the types of cases you handle—do you think any of these factors might predispose the facility to further infections, whether Marburg or something else? Could it put your facility at risk?

R: Yes, I would say so.

I: In what way?

R: The facility is still under development, so there are limitations.

I: I see. Could you elaborate on that?

R: You mentioned bed capacity and structure, right? Well, we still don’t have enough space, and certain branches of the facility are incomplete.

I: So, do you think that puts your facility at risk of outbreaks?

R: Yes, definitely.

ENROLLED NURSE (EN)

|  | |
| --- | --- |
| RESPONDENTS | R |
| INTERVIEWERS | I |

**I:** We want to find out when the Marburg virus was detected in your facility while you were there. What were the administrative processes that went into taking the sample, the protocols implemented, and how were you eventually informed that it was Marburg? Can you describe the administrative steps for sampling and the final report on the test outcome?

**R:** Okay, that day I was on the afternoon shift. When I arrived, they informed me that a patient had been admitted with complaints of headache and vomiting. Later on, the patient began bleeding from the nose. He was referred to Komfo Anokye Teaching Hospital (KATH), and I was assigned to take him there. Just as we were preparing to leave, the patient passed away.

**I:** By "KATH," do you mean Komfo Anokye Teaching Hospital?

**R:** Yes, that’s correct. The patient’s family was saying they didn’t have money, so there was a delay. By the time the ambulance arrived, the patient had already expired. An officer *(name deleted)* and I were the only staff around, so we handled the last office procedures. Even though the patient had passed, he was still bleeding. We managed to stop the haemorrhage before taking him to the morgue. The next day, another patient arrived with similar symptoms, and Dr. ........ *(name deleted)* decided that a sample should be taken to identify the disease. They sent a sample to the lab, and later they informed us it was Marburg. They gathered the staff who had been on the morning and afternoon shifts, along with some doctors, and told us it was Marburg disease. We were informed that we would be quarantined. When we were sent home, we thought they would give us some medicine, but they didn’t. They told us there wasn’t any specific medication for it. Instead, they asked us to stay home, avoid traveling, and gave us vitamin C along with some food supplies. Later, they called us to take samples from all the staff. Five days later, they came back and confirmed that none of us had contracted the disease.

**I:** So, being told there was no medicine for Marburg and only being given vitamin C, how did you feel? Were you afraid?

**R:** I was really scared. I even called my father, and he sent me a COA Mixture for extra protection. My father told my sister to bring it to me, but he also asked her to stay away. One of my tenants would always wear a mask whenever he saw me, and I didn’t even know if Marburg was airborne.

**I:** You must have been really scared. Sorry about that. But during quarantine, whenever you felt any slight headache, did you think it might be the disease?

**R:** Yes, yes, I did.

**I:** Was that constantly on your mind?

**R:** Yes, whenever I had a headache, I wondered if it was the Marburg, and I even started researching out of curiosity.

**I:** How did it affect your social life?

**R:** Like how?

**I:** I mean, being confined to a room, not being able to go out, and not seeing your friends. How did that make you feel?

**R:** I was okay at home.

**I:** What kept you okay?

**R:** I watched movies and did some cooking to keep busy.

**I:** Did your friends visit?

**R:** No, they didn’t. Only my sister came to bring me the immune booster.

**I:** What about your colleagues?

**R:** No, none of my colleagues came by. Only one officer *(name deleted)*, who was also quarantined, brought me something, and that was it.

**I:** How did you feel, realizing that not many people were coming to visit?

**R:** Some people even teased me, especially when they called us to the facility to take our samples. They would say things like, "Don’t come too close, just leave your sample and go." It was like they didn’t want to risk catching anything from us.

**I:** Did fear or anxiety affect your sleeping pattern?

**R:** No.

**I:** What about your eating pattern? Did it change?

**R:** No, it didn’t.

**I:** During quarantine, did you start to rethink your life or think deeply about who you are? Did this experience make you appreciate life differently?

**R:** No, I didn’t feel that way. I was confident I wouldn’t catch it, so I wasn’t very afraid.

**I:** After quarantine, when you returned to work, did it affect how you handled patients or your approach to your job?

**R:** Yes, it did. Whenever I approached a patient’s bedside, I wore a mask and gloves to protect myself. I thought to myself, "If Marburg killed someone, it could kill me too," so I took extra precautions. Even when I was giving patients their medication, I protected myself. My colleagues would tease me, saying, "Oh, you’re always protecting yourself because you were quarantined," and I’d just say, "Yes, I need to."

**I:** Did that make you uncomfortable, or did you feel it was necessary?

**R:** I felt it was necessary.

**I:** Were you comfortable being quarantined at home?

**R:** I’d never been quarantined before, so it wasn’t easy for me. But there wasn’t much I could do about it.

**I:** Feel free to talk about it. How did you really feel about being quarantined for the first time?

**R:** I was okay staying here.

**I:** Were you staying alone?

**R:** Yes, they asked us not to go home, and I was worried that my parents might catch it, so I stayed here alone. It was like how COVID was. Anytime I coughed, I’d be worried they might suspect something.

**I:** So, was watching movies your main way to cope, or did you have other coping mechanisms, like talking to friends or family?

**R:** Mostly, I just spoke with my parents. They’d call to ask what was happening, if they had taken our samples, and what the results were. My father was the one asking me those questions; I didn’t tell my mother.

**I:** Why didn’t you tell your mother?

**R:** I know my mother, and I knew she would be very worried, so I kept it from her. Only my father knew about it.

**I:** Aside from the quarantine and sample-taking, were there any other measures put in place after the Marburg detection?

**R:** No.

**I:** Were there any institutional policies in place that made you uncomfortable?

**R:** No, I wasn’t uncomfortable with any of the policies.

**I:** So, let’s say you were asked to quarantine to avoid contact with others, but you had to go to the hospital to take your sample. Were you comfortable with that procedure?

**R:** Yes, I didn’t have an option, so I went.

**I:** Did you walk, or did you take public transportation?

**R:** I took a car.

**I:** So, assuming you had the virus, don’t you think that could have endangered others?

**R:** Yes, it could have, but I didn’t think about that at the time.

**I:** You mentioned that you expected to be given medication but were only provided with vitamin C.

**R:** Yes, that’s true.

I: Was that an expression of dissatisfaction?
R: Yes, because I thought we would be given medicine, but they only gave us vitamin C. So, I even asked the matron why they only gave us vitamin C, and she said they didn’t have medicine for it.

I: Besides medication, and the issues you mentioned, were there any other concerns you raised to either your colleagues or the in-charge about things you expected them to do or other things they should consider?
R: No.

I: With your workplace, the architecture of the facility, the proximity of different areas, whether they’re connected or not, the medical equipment you use like bed capacity and machinery, and whether your hospital is a referral unit or not, do you think any of these or other characteristics make your facility open to further outbreaks?
R: Yes, our hospital is small, especially the emergency unit. We have only four beds, and the space is limited.

I: So, how do you manage those emergency cases when there are many cases at once?
R: We refer some of them.

I: When treating a patient, if you observe that the case might be infectious but you only have that emergency unit to handle emergencies, yet it's unsafe, how do you handle it?
R: When patients come, and the place is full, we tell them to go to other facilities. During the quarantine, I was informed that they closed the room where the patient had passed away.

I: In terms of the number of staff available to respond to emergencies, do you think the facility has enough?
R: No, the staff is limited.

I: Which type of staff do you think are needed to handle the cases, based on your assessment?
R: We need more nurses, especially in the emergency unit. We are very few; on the day the patient expired, there were only two of us there.

I: Were you there when the patient expired?
R: Yes, I was the one taking him to Komfo Anokye, so it was only one officer *(name deleted)* and me, the staff nurse, there.

I: How did you feel?
R: It wasn’t easy.

I: Or have you seen this happen often?
R: I haven’t seen a patient expire and still bleed, so that was frightening.

I: So, that was what scared you.
R: Yes.

I: When the virus was detected and staff were supposed to work following certain protocols, were you given any training?
R: Yes.

I: What kind of training?
R: For me, it was my night shift, so I wasn’t there when they held the meeting, and I was punished for not attending, even though I was there when the patient expired.

I: With the Marburg virus specifically, how was information communicated to the staff and patients in the facility?
R: The patients didn’t know about it.

I: The patients didn’t know?
R: Yes, because if they were told, they might not return to the facility. I don’t think they would come back, so it was only the staff who were informed.

I: How was the information given to the staff?
R: We have a WhatsApp group.

I: Only through that medium?
R: Yes, but those who were quarantined received a phone call, and we had a meeting.

I: Are you aware of whether your facility has any disaster management plan or a strategic approach to alert staff when such things happen, so they know the standard operating procedures for handling such situations?
R: Yes.

I: Can you describe it? In response to the Marburg outbreak, how was it supposed to be managed?
R: For Marburg, they provided us with overalls, face masks, and we had an isolation room for such cases. When you’re going in, you don’t take items from the ward to the isolation area. We separated some items, so when you go there, you take a nose mask and overall.

I: A well-equipped isolation unit?
R: Yes.

I: You mentioned how samples were taken and sent out for testing, your journey to Komfo Anokye Teaching Hospital, etc. From your observation or what you heard, was the process of taking the sample, submitting it, and getting results back smooth, or were there challenges?
R: They took those who were around to the lab for the investigation.

I: And there were no challenges with that?
R: No.

I: What about the sample that was taken from the patient to be tested for Marburg? Did you hear anything about delays or challenges getting it tested?
R: No.

I: You mentioned that on your way to Komfo Anokye, the patient expired?
R: No, it was when we were about to go.

I: OK, when you were about to leave, and there was another patient showing similar symptoms.
R: Yes, the next day, someone came with the same complaints, and that person was also transferred to Komfo Anokye, and he too expired.

I: Can you describe the process to transfer the patient—like through what medium, from which unit the patient was taken, the evacuation process, and how the area was decontaminated?
R: Like when we were able to transfer the patient to Komfo Anokye?

I: Yes.

I: Was the patient in your ward before the transfer?
R: Yes, that was the isolation room.

I: And then the patient died?
R: Yes.

I: So, what was the decontamination procedure? How was the patient moved, and how was the area cleaned to be safe for others?
R: For the second patient, I wasn’t around, but for the first one, I was there. So, we did the necessary procedures. We soaked the BP apparatus, bedspread, and other items in a chlorine solution for some hours before washing them.

I: Do you have a morgue at your facility?
R: No, we don’t.

I: How did you handle that?
R: The relatives said they didn’t have money at the time, so the patient was there for about two hours.

I: After passing away?
R: Yes, the patient was bleeding, so we used a giving set to control the bleeding, and then we cleaned the body before calling the morgue.

I: Sorry, those who were cleaning the body, were they exposed?
R: I was with one officer *(name deleted)*, doing it.

I: Do you think you were adequately protected?
R: No, no.

I: Can you describe how you were dressed and what you did?
R: When the patient expired, we prepared the body. He had an NG tube, so we removed the tube and the IV line. After removing it, the patient continued to bleed, and his eyes were dilated and black, which was frightening.

I: So, you were really scared.
R: Yes, I’d never seen a patient who expired with eyes like that. We even wondered if it was due to a snake bite or poison because the blood wouldn’t clot and kept flowing.

I: How were you dressed?
R: We were wearing our usual scrub uniforms, surgical gloves, and a nose mask. Unfortunately, our in-charge wasn’t wearing a mask, and some blood splashed on his face. He immediately left the room and later took the patient’s sample to check for hepatitis and retroviruses. Later, they told us it was Marburg.

I: So, the patient was there for two hours before the results came?
R: Yes.

I: And all this time, were other patients in the same ward?
R: Yes, other patients were there. We didn’t know it was Marburg or COVID at the time, so he stayed there until they came for the body.

I: When the information came that it was Marburg, what was done to the other patients in the room?
R: They were discharged, but I don’t know if they were called back.

I: Did you know about any contact tracing?
R: I heard that the patient's relatives were infected and also passed away.

I: Was this the patient who expired, not the discharged ones?
R: Yes, the one who expired, not those discharged.

I: You mentioned having an isolation unit and an ambulance. Did you have enough PPEs and ventilators?
R: Later on, they provided us with more.

I: What other equipment do you think is needed for managing outbreaks like this?
R: We need more PPEs.

I: Which PPE specifically?
R: Overalls and face masks.

I: Anything else?
R: We have surgical gloves.

I: Does the emergency unit have a ventilator?
R: No.

I: After experiencing this exposure, was there any counselling unit set up for staff who were traumatized?
R: No.

I: Did you or anyone else seem to experience trauma?
R: No.

I: Did the Marburg issue affect your personal life, requiring any lifestyle changes?
R: No.

I: So, you didn’t need to sanitize or wash your hands more frequently?
R: I told my colleagues that if we were going to die, it would have already happened, so I made sure to protect myself.

I: Did these changes come after you were quarantined for Marburg?
R: Yes, after.

I: Were you here during COVID?
R: No, I came in May.

I: Thank you. Is there anything else you’d like to share about this experience?
R: No. Thank you.

MEDICAL DOCTOR (MD)

|  | |
| --- | --- |
| RESPONDENTS | R |
| INTERVIEWERS | I |

***Demographics*** *(deleted)*

**I:** In terms of the Marburg disease, how would you describe how it was detected—not necessarily the medical or clinical approach used, but the administrative procedures that were followed after it was discovered that the cases were not normal? What administrative steps were taken to help identify the condition as Marburg?

**R:** First, when we have such a case, administratively we need to inform all stakeholders. For instance, here, I informed my medical supervisor, who then called the district director. They also came around to follow up. So, from an administrative point of view, aside from identifying it, that’s what we did. Afterwards, the whole facility was also informed.

**I:** Did you have contact with any of the cases?

**R:** Yes, I’m actually the one who came in contact with the patient. I’m the one who suspected that it was a case of a hemorrhagic condition. So that’s what happened, and I came in contact with the two patients.

**I:** And regarding the decision to quarantine, what considerations went into determining where the staff would be quarantined, what needed to be done for them, and the number of days, etc.?

**R:** The quarantine period was set at 14 days. Initially, the administration wanted to arrange a place for all the staff who had contact with the patient. However, because the facility couldn’t afford that, we decided on home quarantine. Many of us come from different places, so we rented accommodations from various locations. We were instructed not to travel or go out unnecessarily. To support this, the facility provided the quarantined individuals with food supplies to avoid them needing to go out to buy anything. Additionally, management made regular visits to ensure that they were staying at home and not disobeying orders.

**I:** Aside from unannounced visits to check if staff were still in quarantine, were there any other measures in place to enforce compliance?

**R:** Yes, aside from unannounced visits, there were regular phone calls to check in and monitor how they were feeling and to see if they were experiencing any symptoms at any moment.

**I:** And yourself—how did you feel about coming into contact with a deadly virus, knowing its potential impact? You were also quarantining at home, and since you’re married, your family was affected as well. What thoughts were going through your mind during that period? How did you feel?

**R:** As I mentioned earlier, we all come from different places. My wife and kids are in .......*(name of town deleted)*, and they usually come over on weekends. So at that time, I felt relieved that they were not around and wouldn’t be affected. Even after COVID, which people assume is no longer a threat, I always wear my mask. I felt somewhat assured of my protection because when I approached the patient, I was wearing a mask and all the PPEs that were available. I didn’t feel a strong sense of danger since I was also wearing gloves. There was a nurse who was closer to the patient and had contact with the patient’s blood. He mentioned that something, maybe blood or a fluid, had splashed on his eye, and we advised him to wash it immediately. That served as a barrier, so he was encouraged not to fear. Since it didn’t reach any tissue, I believed it wouldn’t be a problem. So honestly, I didn’t feel too threatened, and I believed that none of us involved would be affected.

**I:** During that time, you say you were “not all that threatened.” Does that mean you felt some level of fear?

**R:** Of course, you know, we weren’t expecting it. When something unexpected comes, you don’t take it lightly. It’s different from something like malaria or stroke that we encounter daily. Something you’ve never thought of suddenly appears, and of course, it crosses your mind. But while thinking about the risks, you also think about what you did right. If you focus on what you did wrong, it may make you anxious, but if you consider what you did correctly, it reassures you. I always say this—people often ask why I continue to wear a mask even after COVID, and I tell them it’s just for my protection. So yes, there was some fear, even if just a little.

**I:** Did that fear affect your sleeping pattern at the initial stage?

**R:** Yes, especially after the samples were taken. I was anxious to hear the results, wondering if the patients would test positive. From the time the samples were collected until the results were announced, my sleep was disturbed.

**I:** So did it affect your eating habits as well?

**R:** Yes, it did. Every call, every message would make me anxious. Once a patient has died, and you’re waiting on results, you can’t help but wonder, "What if it’s positive? What will happen next?" It was a challenging period, but by God’s grace, we managed.

**I:** During the times when you struggled with sleep and your daily life was affected, how did you cope?

**R:** As I mentioned earlier, I kept focusing on what I did right. To distract myself, I watched movies, especially cartoons, to release stress. Sometimes I tried reading, but that could sometimes lead my thoughts back to clinical issues. But even after watching, the thoughts would often come back.

**I:** How did your family help during that period?

**R:** I didn’t inform any of my family members, except my wife.

**I:** Any reason why?

**R:** Yes, because they would have gone through the same trauma. If I had told my mother, brothers, and sisters, they would have called me every minute. So, I kept it to myself and only told my wife. I had to encourage her too; if I hadn’t informed her, she might have wanted to come around. Once the news started circulating, she would have asked me about it. Friends from medical school began calling, saying something happened at our facility, advising me to be careful. I told them not to worry. It was only after our results came back negative that I felt confident enough to inform my family.

**I:** At your facility, aside from calls to check if staff were adhering to quarantine rules, was there any counseling service or support organized for staff feeling traumatized or depressed?

**R:** Are you asking about that time or currently?

**I:** Yes, within that period and immediately after quarantine.

**R:** After quarantine, the medical supervisor met with all of us. Before quarantine, individuals were spoken to individually to avoid alarming people. After the quarantine period, we were addressed collectively.

**I:** Was there any counseling option available for those who felt mentally affected?

**R:** No, but we were told to keep talking to people we knew and, if needed, reach out to the medical supervisor, myself, the nurse manager, or other supportive staff. We don’t have a counseling unit per se, but we have individuals who provide support for cases like these. For instance, patients with HIV regularly receive counseling support.

**I:** How did the quarantine experience and exposure to risk affect you professionally? For example, during COVID, we washed and sanitized hands frequently, but these practices reduced over time. Did anything change for you after quarantine?

**R:** Psychologically, it impacted me and my work—I realized that our work is risky. I learned to take infection prevention seriously. Even after COVID, I still wear a mask. I advise my colleagues to take infection prevention and PPE use very seriously. This experience has motivated me to be diligent about safety practices, as we don’t know when another similar situation might arise.

**I:** Has your frequency of handwashing and sanitizing changed?

**R:** Oh, it’s increased.

**I:** By how much?

**R:** Before, sometimes, I’d see a patient without gloves, but now, if it’s necessary, I wear them. I sanitize my hands and stethoscope between patients, which we were supposed to do anyway, but now I’m extra vigilant.

**I:** Did you have enough PPE and other equipment to work safely?

**R:** Yes, but you know, in Ghana, supplies are often limited.

**I:** Can you explain where the gaps are?

R: The truth of the matter is that sometimes the facility doesn’t have gloves, so this becomes a challenge at times. Occasionally, for example, with sanitizers, it can happen that a section of the department struggles to get some, and these are all administrative issues. But you know, here in Ghana, you can’t speak too much about that. In terms of isolation spaces, we don’t always have enough. This is the district's first referral hospital, so all kinds of cases are sent here, but we don’t have enough rooms for isolation centers. We have an isolation center for every condition, which is just a single place, and it’s not even spacious. So these are all challenging issues that we face when there’s an outbreak of such conditions. Sometimes those personal protective equipment (PPE), isolation centers, and even instruments become challenges. Sometimes, we have to use tools or instruments like blood pressure (BP) apparatus in ways that aren’t ideal. Ideally, we should have an isolation room equipped with monitors, BP apparatus, and other instruments specifically for that room, but we don’t have these things. So, in the first place, this becomes a challenge in our facility.

I: Can you describe your ICU? What is its status, and how does it help you to do your work?

R: Very bad, because if we have to talk about it, if we have any other case like that, we manage them in the isolation room. Like I said, we have to bring the monitors and other things into that room.

I: Do you have an ICU in your facility?

R: We don’t. We manage patients with our tools, especially in the emergency area; that is where we normally manage them.

I: In terms of the facility, is there any standard written procedure within the facility, as you have been working there for some years now, that is usually applied anytime there’s an outbreak or an emergency case? What do you know about any existing standard operating procedures within the facility for when an emergency occurs, such as the Marburg outbreak?

R: Since I started working here, I can say that mostly, if something like that happens, we suspend all cases and refer them. When it comes to surgeries, we suspend everything. That’s the first thing we do—suspend and refer.

I: Can I conclude that there isn’t any established plan with specific procedures that workers follow when there’s an emergency or an outbreak? In other words, can I conclude that there isn’t a standing protocol like that in your facility?

R: Before, we didn’t have anything like that. But now, I think after this Marburg outbreak, there have been some protocols developed for what to do when there’s an outbreak. But before, I don’t know much about that.

I: You mentioned earlier that the staff was called to address the situation when the case was detected, and that included phone calls when you were quarantined. But aside from these methods, what other ways were used by management to communicate the news to all the staff and patients to avoid the spread of the disease?

R: I think we have a facility WhatsApp page, and information was sent there. In terms of the community, the facility went to the local information centers to announce the situation so that people would be aware. We actually went to information centers around, and for those who would be referring cases to us—as I mentioned, we are a referral center—we had to send information to those facilities, the CHPS compounds, and the health centers around so that they would also change their referral center.

I: In terms of the security protocols for people entering and leaving the facility, did that change when the disease was detected? How would you describe it?

R: Sincerely, I would say no; it has not changed.

I: Regarding human resources, do you think a referral center like yours has enough staff, both in terms of numbers and cadre, to manage an outbreak if it were a large-scale outbreak?

R: Our human resource numbers are seriously not enough at all. Aside from the numbers, the expertise of the staff here isn’t sufficient. I don’t know if you’ve been here before, but we are under ....... district *(name deleted)*. We are working, but our numbers are small. This place is somewhat remote, so when people are posted here, they often don’t come. Most of our nurses are enrolled nurses; we don’t have enough diploma or degree nurses. As a doctor, working with this team, you often have to supervise closely to make sure things are done correctly. As a referral center, the work we’re doing here is challenging without the required staff numbers or expertise.

I: In terms of infection prevention, could you describe the procedures you used in evacuating bodies from the facility? How was the condition of the morgue, and how long did it take to move the bodies to your facility’s morgue and disinfect the wards where the deceased individuals were before being moved?

R: When it comes to evacuating the bodies of patients, the truth is that our place here is quite remote, and understanding of health procedures is sometimes lacking among people, which we must address most of the time. The first case came from the north. When the patient expired, we tried to talk to the family, but they didn’t want to understand anything. They took the body to the north. Later, I think one or two relatives were also affected and passed away. The lack of understanding or high illiteracy rate didn’t allow us to manage the body properly. They insisted on taking the body to the north immediately, so we couldn’t handle it as needed. But with the second case, we were able to manage it better. We were able to speak with the family, and they took the body home. The district director also followed up to ensure it was handled properly. I don’t know all the details, but I know the district director was involved with the second case, which was from around this district. So, we could follow up, but with the first case, we couldn’t. In Ghana, they managed to move the body all the way from here to the north without any protection.

I: How long did it take to move the body from the emergency unit, where it was held after the patient expired, to either the morgue or to escort it?

R: For the second case, it might have been about 30 minutes.

I: But the first case took longer?

R: Yes, they likely arrived the next day.

I: So, was the body still in the emergency unit?

R: No, I mean the day the patient expired. She came in the morning, and I think in the evening or afternoon, she passed away, but I don’t know how long it took for them to move the body.

I: Was the deceased patient in the same room with other patients until they were moved?

R: No, before we detected it, there was no other patient in the ward; he was alone. He came at night, and that night there was another patient. But when we assessed the case and realized it was serious, we moved other patients out of that room.

I: Do you have a morgue?

R: No, we use a private one about 30 minutes from here. We really wish someone could build one for us.

I: When ambulances are called to transport a body, are they informed of the likely risk? From your personal observation, do you think they could have exposed themselves during the process?

R: After the incident, they were informed and given some PPEs right away to ensure they didn’t expose themselves. I remember they were informed, and we helped them with all those precautions.

I: Do you have ventilators, testing kits, or a mini-lab for testing?

R: Yes, we have a lab.

I: And ventilators?

R: No, we don’t have ventilators.

I: Are you aware of any policy or guidelines on the working relationship between the Ghana Health Service (GHS) and CHAG?

R: Yes.

I: Regarding the district you are in now, what has been the working relationship between your facility and the district health directorate, as well as other GHS facilities?

R: For us, I’m not aware of all the details, but the district director comes here for any information needed about cases requiring public attention. They oversee public health issues and concerns, and they are thorough, so we work closely with them.

I: When logistics are distributed to GHS facilities, does your facility also receive the same resources?

R: Sometimes they do, but the truth is we don’t receive much from them. It was during the Marburg issue that they planned to bring us PPEs, but even then, it wasn’t much.

I: Have you experienced any challenges in responding to emergencies and disease surveillance, particularly when working with the district health system?

R: No, apart from issues with supplies like PPEs, they respond to us quickly. Sometimes there’s a delay with sample collection, but otherwise, they support us.

I: So, would you say your district health directorate is very accommodating?

R: Yes, I would say so.

I: Are there instances where, if you need to contact the district directorate, you might use informal methods to get their attention?

R: Yes, they communicate with us through WhatsApp and other methods. Aside from the formal channels, they call and come around.

I: Aside from the district directorate, are there other agencies or individuals you collaborate with to handle emergencies or important issues in your facility?

R: Being a CHAG institution, we mostly work with other CHAG facilities and the CHAG secretariat. We also liaise with GHS and occasionally coordinate with the hospitals' regional heads if there’s an issue.

R: Being part of a CHAG (Christian Health Association of Ghana) institution, I believe that we mainly work with other CHAG facilities, individuals, and doctors from various centers. I am not aware of any partnerships with other agencies that we work with.

I: So, you’re not sure whether your facility receives donations or resources from other agencies or facilities?

R: No, I’m not sure.

I: In regard to the Marburg outbreak, aside from the district health directorate that provided you with PPEs, you didn’t receive any resources from other institutions or agencies?

R: I’m not aware of any other resources from outside institutions or agencies.

I: Apart from collaborating with facilities in your catchment area, do you have any other established relationship with a particular facility that you regularly work with to accomplish certain tasks?

R: Yes, we work closely with one Hospital *(name deleted)*.

I: Is that the same as ..... Hospital *(name deleted)*?

R: No, ......*(name deleted)*  is a health center, not a hospital. However, when we have emergency cases or require additional resources, we reach out to ........... Hospital *(name deleted)*.

I: And when you do, what is the usual response from .......... Hospital? *(name deleted)*

R: They are generally receptive, but sometimes they can’t fulfil all our needs, so we end up having to send cases to KATH (Komfo Anokye Teaching Hospital).

I: I understand that one of the patients who was later diagnosed with Marburg visited the ...... Health Center *(name deleted)* for three days but didn’t improve before coming to your facility.

R: Yes, that was the first case.

I: Just to confirm, is this the same ......... Health Center *(name deleted)* you mentioned earlier?

R: Yes, it is.

Thank you.

NURSE OFFICER (NO 2)

|  | |
| --- | --- |
| RESPONDENT | R |
| INTERVIEWERS | I |

Demography: *(deleted to protect the privacy of participants)*

I: Regarding the Marburg case, can you tell us what administrative procedures were taken when you realized that the symptoms were unusual? What steps did you take to get the testing done and announce the outbreak?

Response (R): The patient's name is ......... *(name deleted)* , from ..........*(name of place deleted)* in the ........ municipality *(name deleted)* . He is 51 years old and came to the facility on the 28th of June. By that time, we already had one case on the 26th. So, he came in on the 28th, which was in 2022, with complaints of vomiting and loose, watery stools. It was an OPD case, but he was brought to the emergency side and accompanied by an OPD nurse. They came in, and we welcomed them. The nurse handed over to us, saying it was an emergency treatment issue and that the patient had already seen the prescriber in the consulting room. For emergency treatment, the prescriber asked us to pass an NG tube for drainage and set up oxygen for the patient. So, we made him comfortable on a ready bed, passed the NG tube, and drained the blood fluid from his abdomen. The prescriber also asked us to administer omeprazole, buscopan, and antacid.

I: When you realized that what you were observing wasn’t normal and that you needed to go beyond your facility’s capacity, what administrative procedures were put in place? How did you coordinate to get the sample out, decide to involve other departments, and eventually detect that it was Marburg?

R: Like, what we did for him before realizing we had to take a sample for investigation?

I: Yes.

R: The doctor asked us to give him one unit of blood and later requested some lab investigations, which included the FBC, urine array, and DUU.

I: Whose decision was it to send the sample out for testing?

R: It was Dr. Ata's decision.

I: Was that process smooth? How was the sample taken out, who was it given to, and what happened afterward with the staff who had contact with the patient?

R: After taking the sample for investigation, the staff members who were involved were quarantined for two weeks.

I: When you learned that it was Marburg disease, did the news affect your daily life or medical practice? What changed after you heard that what you had worked on was Marburg?

R: When I was called by the MO and NO to break the news, I felt very down. Knowing that I had worked on the patient, I feared that I might have the virus, so I was very low.

I: How did they break the news to you?

R: The MO called us, and we went to his office. He told us the result and explained the investigation.

I: What measures were put in place when they realized it was Marburg?

R: In the emergency department, we have two rooms. They quickly turned one room, which has three beds, into an isolation unit. We were given PPEs to wear, and we were instructed to wear them whenever attending to the patient.

I: You mentioned feeling sad and scared, thinking you might have contracted the virus. Did that feeling affect your work or your relationships with others?

R: Yes, it affected my work because I became very conscious when attending to patients. I didn’t know which patient I would be exposed to.

I: So, were you washing your hands after attending to each patient?

R: Yes, after every procedure, I washed my hands and wore gloves and a nose mask everywhere I went.

I: At that point, I’m sure you were expected to wear PPE at all times. How did it feel to go through that again, especially right after COVID? What was your experience?

R: I wasn’t around during the COVID-19 period because I was in school, so I didn’t experience wearing PPE then. This was my first time wearing it. Wearing it around is not easy because of the heat; it’s uncomfortable, but I had to wear it.

I: How did this affect your relationship with other staff and your family? You have kids, and you were supposed to stay away from them for two weeks during quarantine. How did that impact your social life with your family and colleagues?

R: My family isn’t with me; they are in ........... *(name of town deleted)* , and I am in .............*(name of town deleted)* , so I am not with them.

I: Were you quarantined at that time?

R: Yes, I was quarantined for 14 working days.

I: At home?

R: Yes, at home.

I: During the period when Marburg was detected and immediate actions were enforced, were there any institutional policies that you felt uncomfortable with? Were there things done that you thought shouldn't have been?

R: No.

I: Did you feel the support system from the facility was adequate?

R: I would say it wasn’t adequate because the emergency room is small, the PPEs were not enough for all staff, and there aren’t enough staff members in the emergency unit. We are about 15 staff in total.

I: In terms of your family, what kind of support did you receive from them during that time? How did you break the news?

R: I didn’t tell them because they wouldn’t take it well psychologically. They might have come over to see if it was really true.

I: Was it also because you didn’t want them to come into contact with you?

R: Yes, so up to now, they don’t know about it, even afterward.

I: Now that you know you’re okay, have you thought of telling them?

R: Yes, but they might still think the virus is present.

I: At the time when you were feeling afraid, how did it affect your sleep pattern?
R: You would sleep, but in the middle of the night, you would wake up and start thinking about all the signs and symptoms. When you urinate, you check if there’s blood, and when you defecate, you check if there’s blood before you flush. So, it wasn’t easy.

I: And your eating pattern?
R: I was very careful with my eating pattern. I was eating more vegetables and making sure I included fruit as well. So, I was very cautious about that.

I: So, when you were quarantined, were you anxious or afraid? How was the feeling?
R: The feeling was mixed for me. On one hand, I was happy because I was able to rest and didn’t have to go to work. But, on the other hand, I was not happy because I knew I had come into contact with a patient. I also wasn’t able to relate with my colleagues or call my family, and that part wasn’t good.

I: So, you weren’t relating to your colleagues and weren’t calling your family. How were you able to cope?
R: I was calling those who were involved. We would talk to each other and encourage each other. So, every day we would call one or two times to check on each other and make sure no one had developed any signs or symptoms.

I: Did you decide to quarantine at home?
R: No, it was decided by our Medical Officer (MO) and nurse manager.

I: What is your impression about quarantining at home for such a disease?
R: I became very conscious of my daily activities regarding any outbreaks.

I: During COVID-19, there were isolation centers where people were quarantined, but with this situation, you weren’t given an option to quarantine elsewhere. You were told to quarantine at home; is that correct?
R: Yes.

I: Regarding your work environment, do you feel that your facility’s design, equipment, and the roles within the facility increase the risk of outbreaks?
R: Yes.

I: In what way?
R: Because we have one building that contains all our departments in a single, non-story building.

I: How do you manage?
R: So, if there is an outbreak, it’s very easy for it to spread.

I: Are you aware of your facility having an internal emergency and disaster management plan or any existing document, plan, or strategy on how to manage emergencies or disasters like this?
R: We have been provided with PPEs, and we have two units for emergencies. We use one side for isolation, so in case of an outbreak, we have an isolation room for such cases.

I: We know there are standard operating procedures (SOPs) for certain things. For example, during COVID-19, there was a protocol to follow. Is there an existing protocol for all emergency cases, which can be adapted to different types of emergencies?
R: We were given a chlorine solution to disinfect all our surroundings. When a car brings a patient to the facility, once the patient comes out, we disinfect the car to prevent any infection.

I: What kind of communication structure existed during the outbreak? How was information shared among workers and patients when the disease was detected?
R: For the patients, our Outpatient Department (OPD) educated them on Marburg every morning, and we had posters about Marburg.

I: To protect both patients and staff, in addition to the quarantine and awareness campaigns, what other measures were put in place to ensure the facility’s safety?
R: We avoided wearing gloves to open doors and constantly disinfected doorknobs, chairs, desks, and other surfaces. We were also closely monitoring for serious cases.

I: Did you receive any training on managing outbreaks from your facility, CHAG, or GHS, specifically for Marburg?
R: Yes, we had training with the nurse manager. They taught us how to handle such cases, particularly how to wear PPEs before attending to patients. We had a short workshop on how to properly wear the PPEs.

I: Regarding staff strength, do you think you had enough personnel or specific cadres of staff to manage the outbreak effectively? What was your impression of the staff strength at the time?
R: We were short-staffed during that time because seven of us went into quarantine. The nurse manager had to bring in staff from other units to help those who remained in the emergency unit.

I: Which units helped with that process?
R: The OPD and the general ward provided assistance.

I: So, you would say that your human resource capacity is not adequate to handle such situations?
R: Yes.

I: When it comes to sample collection for testing, which agency do you work with?
R: We work with the Disease and Control Unit at ....... Health Center *(name deleted)* . We call them first, and then the sample is taken to Nuguchi for investigation.

I: Were there any challenges during that process?
R: Sometimes, the lab technician would take the sample, but the director or the disease control personnel would not pick it up promptly. So, the technician had to store it in a refrigerator until they could come for it the next day.

I: Regarding human resources, when it comes to priority issues, how is the collaboration between staff and departments to get results, based on your experience with Marburg?
R: Everyone was engaged, and everyone wanted to help.

I: Could you briefly describe the evacuation and decontamination process used during the Marburg outbreak? Were any specific standard operating procedures (SOPs) followed to ensure the area was safe for reuse?
R: We disinfected everything used on the patients with a chlorine solution.

I: Were you satisfied with the process? From your observation, do you think it could have been done better?
R: Yes, it could have been done better.

I: In what ways?
R: If we had more space.

I: You mentioned earlier that seven of you were quarantined.
R: That was just on the nursing side. Including the prescribers, we were about fourteen, with seven being nurses.

I: Based on your over ten years of experience at this facility, which devices or equipment do you think are needed but unavailable to prevent, detect, or respond to outbreaks?
R: We have only one monitor for patients. We don’t have enough thermometers either, and since I don’t work in the lab, I can’t speak for them specifically, but I think they are also short on supplies.

I: Do you have enough ventilators? If so, how many?
R: We don’t have any at all.

I: During COVID-19, were patients referred to your facility?
R: I wasn’t there, so I can’t say.

I: How about the ambulance system? Was it functional?
R: We used our own ambulance as well as the national one.

I: Do you have enough trained technicians for managing emergency cases?
R: We don’t have any emergency nurses; those working in the emergency are regular nurses, not specifically trained for emergencies.

I: Do you think your ICU is reliable?
R: We don’t have an ICU. Our emergency unit, with a capacity of seven beds, handles all ICU cases.

I: Do you have enough testing kits to manage a widespread outbreak?
R: During COVID-19, I was on study leave, so I don’t know.

I: When all of this was happening—being quarantined, facility inadequacies, and low human resources—how did you manage to keep your mental well-being intact and continue doing your work?
R: I tried to stay calm and focus on work to take my mind off the fears of catching the outbreak.

I: Is there a counselling unit or support available for workers dealing with mental stress or anxieties related to outbreaks?
R: No, there isn’t any.

I: So, if you experience anxiety, you have to manage it yourself?
R: Yes, you have to manage it yourself.

I: Is that easy?
R: It’s not easy; it takes time to manage and feel free from it.

I: During the management of the outbreak, was there a time when you or the facility had to rely on informal relationships or processes to get things done quickly?
R: No.

I: Do you have any questions for us?
R: Is there any motivation or recognition included in this study?

I: Unfortunately, there isn’t any, but the findings from this study may help gather support for your facility.

I: Thank you.

NURSES OFFICERS (NO 3)

|  | |
| --- | --- |
| RRESPONDENT | R |
| INTERVIEWERS | I |

I: Can you hear me?

R: Yes, I can hear you.

I: We are discussing your experience with quarantining, how it affected your relationship with colleagues and patients, and the equipment available in your facility, as well as the architectural environment that could potentially expose the facility to outbreaks. But the internet is not really helping. Let me start from that question again. In terms of your facility, as I mentioned, we’re looking at the building construction, design, whether it's compact or spread out, if there’s enough space or not, and if your facility or referral unit has aspects that could make it vulnerable to further outbreaks. Do you believe any of these factors might put your facility at risk?

R: Oh yes, last time I explained that the facility is still under development, so we don’t have enough space, especially with bed capacity and the existing structures. When I was quarantined, nobody called to even ask of my feelings.

I: And I think we were also talking about human resource capacity, and I asked if you’d received any training on responding to an outbreak like this. So, after the Marburg disease preparations, were the staff trained to handle such situations? What kind of training did they provide?

R: They organized a workshop on handling such cases. I think the Infection Control and Prevention (IPC) team came around to train us and the available staff, covering the items we might need if we suspect another case.

I: Did that include any sort of program or activity like a demonstration on handling such situations?

R: Yes, please.

I: Okay. And during the training, did they highlight any equipment that you would need but which isn’t available in your facility?

R: I don’t think so. We had everything, but it wasn’t enough. A few overall suites and gowns, a small emergency unit, no isolation centre…There were small items missing, like extra staff we might need if we suspect another case or if we actually have a case. It wasn’t abundant.

I: Do you have specific names for any of those items?

R: Yes, they brought us some overall suits. Since one of the emergency rooms was used as an isolation room, it has two sections like wards. However, it doesn’t have sufficient bed capacity—each room has only three beds. They took one room as an isolation ward and trained us on how to put on and remove the suits to protect ourselves and others from infection in case of another case.

I: When you compare the training you received with the actual practice, were there challenges in executing what you learned?

R: You mean challenges with executing the tasks we learned about?

I: Yes, exactly.

R: Oh, as I mentioned, the items or equipment they provided weren’t enough. For example, if we have ten staff members in the emergency unit but only two or three gowns, it means we would have to select only certain people to go to the isolation ward.

I: Yes, I understand.

R: So, it wasn’t enough. And considering these items are disposable, it felt like we had to reuse them, even though that shouldn’t be the case. If I wear one, I shouldn’t have to pass it to someone else for next time. This approach could compromise infection control. Also, disinfecting the body suits became an issue since we didn’t have enough. I found that to be a major challenge.

I: Regarding sample transfers, transporting samples taken from cases for testing and returning them, during one of the training sessions, some people mentioned issues with mismanagement, particularly around transferring samples from facilities to district offices and back. What challenges did you encounter when transferring samples for testing?

R: There has always been an issue, even with referral cases. Sometimes getting an ambulance is difficult. The hospital only has one ambulance, and it might be out on another referral. Calling the national ambulance can also be challenging. Even if we reach them, it’s hard to get them to come on time—they make excuses. Though they eventually come, it’s rarely on time.

I: That brings me to another issue. In our last conversation, you mentioned that you don’t have a morgue to hold people who have passed away, even though preventing infection spread is crucial. How did you manage the evacuation and decontamination of exposed areas?

R: Since the hospital doesn’t have a morgue, after the body was removed, we used a 0.5% chlorine solution to disinfect the area and then cleaned it afterward.

I: What’s your impression of that process? Do you think it’s good enough?

R: Based on what I’ve learned and experienced—this being my first time dealing with Marburg—I know that’s the standard method for decontaminating. But for a highly infectious disease like Marburg, I thought there might be a more specific way, maybe involving some form of spraying or other advanced method. I’m not sure, but I was thinking something more specialized could be done.

I: Were you around during the COVID-19 period?

R: No, I wasn’t.

I: Okay. I wanted to know if it was the same chlorine solution used then, but since you weren’t there, that’s fine. Do you have an ICU unit?

R: No, we don’t.

I: So you only have the emergency unit.

R: Mmm-hmm.

I: Does it have proper ventilation?

R: We only open the windows for ventilation.

I: My final question is about the coping mechanisms you used to manage anxiety during the COVID-19 outbreak and the fear of potential infections. We talked yesterday about speaking with colleagues, watching TV, and other measures. Out of these coping mechanisms, which ones didn’t particularly work for you?

R: I think watching movies. Though I tried to use it as a form of distraction, it didn’t always work.

I: Finally, I’ll break this last question into two parts. What advice would you give to facility management to help workers deal with trauma or anxiety from emergency situations like this outbreak?

R: You mean advising management?

I: Yes.

R: I would advise them to check on their staff in such situations, offering reassurance. Even without big promises, talking to someone helps people feel cared for. For example, if I got infected, it would mean a lot to know I have support from the facility. I’m just being honest because there were some things they didn’t do. After we were quarantined, nobody called to ask how we were feeling. They only came by two or three days later to drop off a few food supplies, but no one checked in on us during those two weeks. Honestly, I felt let down, but I tried to brush it off. I was expecting at least the nurse manager or someone in charge to check in or to create a group where we could share experiences. That would have eased some of the anxiety. But nothing like that was done; we were just indoors until the two weeks were up. When we were quarantined, nobody called to even ask how we were feeling. They only came around two or three days into the quarantine. After we came to the house, they came to check on us and brought us a few foodstuffs. That was all till the two weeks were over. Honestly, I felt so bad about it, because I was expecting someone like the nurse manager or the in-charge to call and ask us how we were feeling or if we were experiencing anything

I: I’m sorry to hear that.

R: Oh, it’s okay.

I: Do you have any questions or comments you’d like to share?

R: No, I don’t have any questions.

I: After coming out of quarantine, has this experience affected you professionally in any way?

R: For me, it hasn’t affected me.

I: So, for example, washing your hands after attending to each patient is still part of your routine?

R: Yes, as a nurse, I wash my hands regularly even when I’m at home. It’s not because of Marburg, but just part of our usual routine.

I: Thank you very much.

R: You’re welcome.

I: If we need any follow-up or clarification, we’ll reach out.

R: Okay.

I: Thank you very much, and have a good evening.

R: Thank you...bye-bye.

NURSE OFFICER (NO 4)

|  | |
| --- | --- |
| RESPONDENT | R |
| INTERVIEWERS | I |

**I**: Thank you for agreeing to talk with us today. We’re trying to understand what it was like for you and your colleagues during the recent Marburg virus outbreak. Could you start by telling us a bit about yourself—your age, work history, and family?

***R****: .......Demographic information deleted to protect participant’s identity*

**I**: Thanks for that. Now, when the Marburg virus was detected in your facility, how did you first hear about it, and what was your initial reaction?

**R**: I remember feeling pretty shocked. We’d had patients with symptoms that didn’t look typical, like vomiting blood and diarrhea. When the results came back confirming Marburg, management quickly held a meeting to explain the situation and discuss precautions. My first thought was about my kids at home. I kept wondering if I might have been exposed without knowing.

**I**: What steps did the facility *(name deleted)* take immediately, and how did they communicate these to you?

**R**: After the meeting, they directed us to wear PPEs whenever we were in the ward, especially near suspected cases. They converted one of the rooms in the emergency department into an isolation area to minimize exposure. They also reminded us about hand hygiene, disinfecting surfaces, and wearing gloves and masks. Anyone who had been in contact with Marburg patients was required to quarantine. Management tried to keep everyone calm, but we were all anxious.

**I**: How well did you feel supported in terms of supplies like PPE? Was there enough equipment?

**R**: Honestly, it was tough. We had basic supplies like gloves and masks, but not enough full protective suits. I remember feeling worried because we had to reuse some PPEs due to shortages, which wasn’t ideal. Reusing PPE doesn’t fully protect us, and it made me feel more exposed.

**I**: Ok. So, once you were sent into quarantine, what was that experience like? How did it impact you emotionally and psychologically?

**R**: Quarantine was quite hard. I had to isolate at home for 14 days, which felt very lonely. I was constantly worrying about symptoms. I’d overthink every little ache or pain, wondering if it was Marburg. It even affected my sleep; I’d wake up in the middle of the night thinking about it. It was very stressful, and I felt cut off from everyone.

**I**: How did you manage to cope during quarantine? Was there anything that helped you get through it?

**R**: I kept myself busy. I watched movies, read books, cooked, things that would help me stay distracted. But what helped most was talking with my colleagues who were also in quarantine. We’d check in with each other every day, just to see how everyone was feeling. Knowing I wasn’t alone made a big difference.

**I**: It sounds like that support was important. Did you share what was happening with your family, or did you keep it to yourself?

**R**: I didn’t tell them. I didn’t want them to worry, especially my kids. They might have wanted to come see me or just worry a lot, so I kept it to myself.

**I**: Once you returned to work, did the experience of dealing with Marburg change how you approached your job?

**R**: Yes. I became extra cautious. I washed my hands a lot, kept my mask on, and was careful even with patients who didn’t seem sick. I found myself on edge, especially around patients with symptoms. I didn’t want to take any risks.

**I**: I understand. Was there any support for you and other staff members when it came to dealing with the mental strain of the outbreak? Like counseling or something similar?

**R**: Well, there wasn’t in the facility *(name deleted)*. I feel that having access to a counselor or even just check-ins from management could have helped. Just having someone to talk to about our fears would have been a relief. But there was no real support system, so we just managed as best we could.

**I**: That must have been difficult. Thinking about the facility itself, do you feel that the layout or design of the hospital had any impact on how the virus spread or was contained?

**R**: Yes, our layout made it harder to control the spread. We’re *(name deleted)* still a developing facility and don’t have a proper isolation unit. We converted one room to isolate cases, but all our departments are quite close together, which makes it easy for infections to spread.

**I**: Were there any other equipment or resources you feel were missing that would have helped?

**R**: We needed more PPE, especially protective suits. We also lacked things like monitors, thermometers, and ventilators. These resources would have been useful, especially if more cases had come in. Without enough supplies, we felt vulnerable.

**I**: Looking back, were there any procedural issues or delays that you think affected safety or could have been handled differently?

**R**: Yes, sending samples for testing was difficult. We often faced delays with the ambulance service, which was usually busy with other emergencies. Getting quick results is critical, so these delays made us more anxious.

**I**: If you could make recommendations to management on how to handle future outbreaks, what would you suggest?

**R**: I’d suggest they *(name of facility deleted)* keep in touch with staff in quarantine maybe just check in every couple of days. It helps to know you’re supported. And I’d recommend having proper mental health support. Dealing with these kinds of outbreaks takes a toll on us. We need PPE and basic supplies, but we also need emotional support to get through it.

**I**: It sounds like having that connection and support would make a big difference. Do you feel that this experience changed the way you see your role as a nurse?

**R**: Yes, it has. I’ve always cared about protecting my patients, but now I see how important it is to protect myself too. It reminded me that our own health is just as important because if we’re not healthy, we can’t help others.

**I**: Thank you for sharing that. Did this outbreak experience change anything in your personal life, especially with your family?

**R**: Yes, it made me value my time with the facility *(name deleted)* even more. It also made me more cautious about bringing anything home from work. I’m more careful in general because I see now how easily these things can spread.

**I**: Thank you so much for being open with your experiences. This will help us understand what healthcare workers went through during the outbreak.

**R**: Thank you too. I hope this helps make things better for all of us all.

**I**: We will communicate your concerns.

ADMINISTRATOR (HA)

|  | |
| --- | --- |
| RESPONDENT | R |
| INTERVIEWERS | I |

**I:** We want to understand the kind of relationship your facility has with the district health directorate.

**R:** We have a very positive working relationship. The district director has been supporting us in all our activities. He comes regularly for meetings, discussions, and, if there are challenges, he helps us address them, so overall, it has been a positive relationship.

**I:** However, other CHAG facilities have often complained that when it comes to distributing items and making certain decisions, priority is usually given to the GHS facilities. But from the feedback you're giving, it seems your situation is different. Is there anything specific that your facility is doing that might be influencing this positive relationship?

**R:** Since I arrived here in 2009, I paid a courtesy visit to the district director, and from that time, I have maintained regular contact. I call occasionally just to say hello, and I make an effort to attend meetings. I also encourage my staff in the public health department to reach out. Even when I’m not called, if I have the opportunity, I stop by to greet the team having the meeting and to observe what's happening. When there are discussions related to vaccination or other public health initiatives, we participate and attend performance review meetings. We also provide necessary data as a district facility. This ongoing engagement has fostered a cordial relationship and helped us contribute meaningfully.

**I:** What about the GHS facilities within the ........ District *(name deleted)*? Do you have any kind of relationship with them?

**R:** Yes, we do. Sometimes in a working relationship, you may encounter one or two challenges, but whenever these arise, we try to reach out to the staff involved. In cases of emergencies, especially maternal cases, they refer patients to us, so it's prudent to maintain open communication. If there are complaints about certain behaviours, we follow up and discuss the situation with the staff involved. This approach has contributed to a cordial relationship.

**I:** I understand that the Marburg cases detected in your facility involved one patient who had previously visited the .... Hospital *(name deleted)* three days earlier. When he was getting better, he came to ...... hospital *(name deleted)*, which is under the ....... Government Hospital *(name deleted)*. Is ........ Hospital *(name deleted)* a GHS facility?

**R:** Yes, it’s under GHS.

**I:** So when the Marburg case was detected in your facility, do you know if the ........ Hospital *(name deleted)* was informed about that specific patient who had been to their facility? Were any individuals who had contact with the patient quarantined?

**R:** The facility is connected to the district directorate, so when the protocols were discussed with the district director, I was informed that the director also met with the team and established protocols for them. Therefore, communication was effectively managed on their end.

**I:** Regarding the relationship between CHAG and GHS facilities, do you know what kind of collaboration or policies exist between the two organizations, especially for handling emergency cases? Since you have experience as a public health officer, could you explain any existing guidelines or policies?

**R:** I believe the Church *(name deleted)* and CHAG have a positive working relationship. We often participate in GHS programs when invited. Last year, for example, GHS, ....... (or CHAG), and the Ministry of Health (MoH) organized a meeting, where the Director of Nursing at MoH collaborated with us to draft some policies for nurses. This indicates a positive and cordial relationship.

**I:** When Marburg was detected and you had to coordinate with the district health directorate, which specific offices did you work with, and how did they respond?

**R:** We have not encountered any issues with our district. We mainly work with the district director himself and the disease control office. When we called them, the disease control officer arrived with his team to discuss the situation with the medical staff. Depending on the program, the public health nurse also visits frequently. If we need to invite them to any meetings, they attend. So, I would say we work with all the relevant offices based on the needs of the situation.

**I:** Can you mention some specific offices that you worked closely with and what they actually did for you? For instance, you mentioned working with the disease control officer in the district.

**R:** Yes, it was primarily the disease control team working with the biomedical scientist to send the samples. This setup has been in place since the COVID outbreak. Whenever we had samples, the disease control officer would bring the forms, we’d complete them, and they’d handle the submission. That relationship has remained consistent.

**I:** You mentioned earlier that during the COVID outbreak, you received donations in terms of PPEs and other supplies from the district health directorate. Regarding Marburg, did you receive any tangible or intangible support, such as donations of money or other resources, from the health directorate?

**R:** For the Marburg situation, let me just say that the regional health director actually came to our facility to meet with the management team. The National Public Health Department also visited and guided us to prepare a logistics request through the district, which provided us with a few essential items for managing the situation, specifically PPEs.

**I:** Did the district health directorate participate in any contact tracing efforts? If so, what role did they play in those activities?

**R:** Yes, the district was involved in contact tracing.

**I:** Who specifically from the district handled the contact tracing? Was it the community health nurses or other personnel?

**R:** It was the public health department together with our team of community health nurses. The district health directorate has community health nurses as well, so they joined forces with our team. We conducted training before going out for the contact tracing.

**I:** At any point, did you have to rely on informal relationships? For example, did you need to leverage any informal channels, such as individuals or external units, to get things done during the Marburg outbreak?

**R:** It’s challenging for me to differentiate between formal and informal relationships in this context. We have always had an open line of communication with the necessary teams. For instance, the medical director was in constant contact with both the national and regional teams, discussing cases. So, it would be difficult for me to pinpoint where exactly it was formal or informal, given the mutual and cordial relationship we share with all the teams we work with.

**I:** For instance, if you needed PPEs, did you ever have to use someone who had access to certain offices to ensure the goods were released, like bypassing formal protocols? Or did you perhaps need to approach community leaders when it came to involving the local population?

**R:** No, we have not faced that challenge. Perhaps our director here operates differently from others. I am aware that some facilities have encountered similar issues, but we haven’t had to resort to informal methods to secure items for management.

**I:** Do you have any questions or additional information you think we might have missed?

**R:** I heard that there’s ongoing research on Marburg, but I haven’t asked about how we will receive the feedback. We’ve had instances where people conducted research on various indicators, but we never received any follow-up information. So, I am curious to know how the feedback or outcomes will help us as professionals.

**I:** Once everything is finalized, we will send a copy of the findings to the facility. We will also relay any necessary information to relevant personnel. Since it’s an in-house study, the results of our analysis will be taken into consideration, and any required adjustments will be made to address identified challenges.

**I:** Thank you very much.

MEDICAL DIRECTOR (MDir)

|  | |
| --- | --- |
| PARTICIPANTS | R |
| INTERVIEWERS | I |

*Demographics were deleted to protect the privacy of participants*

I: On the detection of Marburg, aside from the clinical processes that went into taking the samples and testing to identify that this was a new disease you were dealing with, can you share a bit about how the administrative processes went? Specifically, the submission of the sample, the challenges you faced during that process, and when you realized it was Marburg.

R: The first case had been admitted here and was initially detained due to symptoms of gastroenteritis, including diarrhoea and vomiting, along with bleeding. That was the first case. Within that period, the first case unfortunately passed away on the 27th, and I was informed about it. At that point, we decided to take samples because we thought it might be a zoonotic illness, but the patient passed away. Then, when the second case came in with similar symptoms, we thought, “No, this is too much of a coincidence.” So, we took another sample, informed the district director of health, and alerted the emergency team that we might be dealing with a possible haemorrhagic fever, though we were not certain. Initially, we suspected Ebola, so we followed standard infection and control practices. The district used a biomedical scientist to take the sample. The challenge arose after the initial sample collection—it took over six days for the results to return.

I: Were there any issues related to the logistics of handling the sample?

R: According to the district health directorate, they were primarily responsible for transporting and following up on the sample. We took the sample and handed it over to the disease control officer and the district health directorate for follow-up. That has been the protocol since the COVID-19 pandemic: we take samples and then give them to the district health team, who send them to KCCR and later relay the results to us. However, for this case, when we contacted them, they mentioned that they had to send the samples to Noguchi instead, and securing the funds for transporting the samples to Noguchi required coordinating with the regional health directorate. I can’t recall exactly when the samples reached Noguchi, as I didn’t have that information. I remember, though, that about seven days after the samples were collected, the district director called and informed us that the cases had tested positive. Prior to that confirmation, although we had already alerted the emergency staff to follow infection prevention protocols, they were not fully isolating due to the lack of results. Once the positive results came in, the district director informed me that the region was organizing a Zoom meeting to confirm the findings. At that point, we had confirmation that both samples tested positive, and we then proceeded with full isolation and quarantine measures. So, that’s basically what happened prior to initiating full isolation.

I: When the decision for self-isolation was made, were there specific measures put in place to prevent the spread from the perspective of staff who were isolating at home? For instance, were they allowed to go out to buy food or interact with others?

R: Fortunately, the experience with COVID-19 served as a valuable learning opportunity for us. During COVID-19, some staff members had been exposed, and a few even got infected. As a policy, the hospital established a fund or package for staff members exposed at work who needed to self-isolate. We would provide them with groceries and a small cash donation to encourage them to stay home. Myself, the nurse manager, and the district health director would also call them regularly to remind them of the importance of remaining at home. They were given clear instructions not to travel out of the area, and any violations would be dealt with severely. Every time we called, they were at home, although I can't say with absolute certainty that they never ventured out. We provided them with all the support we felt they needed to stay at home and interact only with their immediate family. One of the challenges, however, was that some of them lived with family members and had no other place to go, so isolation was done to the extent possible within the confines of their homes. We also monitored any of their family members who might have been exposed.

I: Reflecting on the COVID-19 experience, with the associated fears of exposure and the anxiety of possibly contracting the disease, was there any form of support to help them cope with mental health issues? For instance, did quarantine include psychological support?

R: Yes, during COVID-19, there was a real fear of mortality. We created a peer support group for those who had to self-isolate, where they could support each other. Professionally, we didn’t have access to psychological help, but we used the peer support system that was put in place during COVID-19. The emergency staff who went through both outbreaks relied on this peer support group, and if anyone had a concern, they would call the nurse manager or me for help. Although there was some suggestion from the regional health to set up external psychological support, that idea never materialized.

I: Regarding mental health, did any of them speak to you about personal challenges related to mental health during the isolation period? For instance, did they mention struggling with sleep, appetite, or focus on their regular duties?

R: No, there was no such report. The main concern among the staff was the fear of testing positive. Many of them were eager to get tested for Marburg, especially during the initial stages of the outbreak, but the protocols for screening hadn’t been fully implemented. In the first week, when they had to self-isolate, disease surveillance protocols hadn’t yet reached the facility level, which meant screening wasn’t available for them. Eventually, however, district surveillance and health teams began to conduct screenings for community members and exposed individuals. We managed to schedule our staff members who were self-isolating to come in at various times for testing.

I: With the testing, what criteria were used to determine who was tested, especially given that there might have been contact with security staff before entering the facility?

R: Testing was based on the level of exposure, and the staff were categorized into three groups. The first group included those with the highest risk, like doctors and physician assistants who directly handled blood and bodily fluids. They were involved in procedures like suturing the first patient and inserting an NG tube for the second one. The second group consisted of nurses who assisted in placing patients in beds and taking vitals. The third group included any other emergency staff. Only the first two groups were screened; the last group was not, as we prioritized those with the highest degree of exposure.

I: For contact tracing, what framework was used to determine the extent of the tracing?

R: Contact tracing was managed by the district health directorate. They gathered contact information for both staff and patients and conducted follow-ups within the community. Even contacts of exposed staff were followed up by the district health team.

I: Were specific people contacted at the facility, or is the facility not directly involved in that information?

R: The immediate family members of the two cases were contacted, including their household members. One of them had come from .......*(name of village deleted)*, so the district health director worked with the ..... health directorate *(name of the same village deleted)*  to reach the family and collect samples. They focused on people who had close contact with the two patients.

I: Was the hospital they had visited prior to coming to your facility involved as well?

R: Yes, that hospital was the ..... Health Center *(name deleted)*, where the district health directorate is based, so they were included in the tracing process.

I: In terms of disease control, information sharing is critical. How did you relay information to the staff and the in-patients?

R: For the staff, we organized a meeting initially for the emergency team to inform them of the positive test results. We reassured them that since they had been using PPEs and following infection prevention practices, their risk was low, but we still advised caution. For the general staff, we held a meeting where we informed them that two cases had tested positive for Marburg. This transparency helped staff from other units stay informed and prepared.

I: What about patients?

R: Rumours had already started circulating in the town that the patient had Ebola. To address this, we organized daily health talks during our morning devotions to clarify the situation, educate staff about Marburg, and dispel any misinformation. We also coordinated with the district health directorate to engage with the community by visiting information centers and churches to provide accurate information about Marburg and its risks.

I: Does your facility have an existing standard operating procedure for managing disease outbreaks like Marburg, COVID-19, and Ebola?

R: At that time, we did not, but we have since developed one.

I: So, you had to react based on the situation as it unfolded?

R: Yes, exactly.

I: In terms of human resource capacity, would you say your facility had enough staff to manage the Marburg outbreak?

R: No, we were stretched thin. We had three permanent medical officers and two physician assistants who were the primary prescribers. When Marburg emerged, one of the medical officers was on leave, and the remaining medical officer and two physician assistants were exposed to the virus, which required them to self-isolate. I was involved minimally, so I didn’t need to self-isolate, but the emergency staff were all impacted. As a result, we had to reach out to CHAG and the regional health directorate for support. Within the facility, we also faced limitations—our resident disease control officer was a field technician rather than a trained disease control specialist. Additionally, our public health nurse had recently graduated, so we lacked specialized expertise in public health.

I: And what about the evacuation, decontamination, and other safety procedures?

R: We don’t have a dedicated isolation area.

I: Yes, and a mop as well.

R: Yes, we don’t have a mop.

I: So, how did you manage the person who passed away until they were taken out? What was the evacuation process and the safety procedures to ensure that the deceased person didn’t …?

R: So, with the first case that passed away, we initially suspected it might be a hemorrhagic illness. Therefore, after the passing, we took as many precautions as possible, following infection prevention control practices to try to decontaminate the area. During the transportation of the body to the morgue, we alerted the morgue and informed them of our suspicions. We told them that we had collected samples and, as much as possible, advised them to use a body bag from the district health directorate and to keep the body as secluded as space allowed. However, at some point, communication broke down, and we later discovered that, after the test returned positive, the body had already been transported to Wa *(a regional capital city in Ghana),* where the family had conducted the final funeral rites without informing us. I believe the fact that this was a private morgue contributed to the challenge. If it had been a public or hospital morgue, we could have insisted on keeping the body for public safety reasons until a final determination was made. But since it was a private morgue, we didn’t have that authority, so the private morgue released the body without informing the district health director.

I: To the family?

R: Yes, to the family. So, by the time we received the positive test results, the body had already been buried in Wa.

I: Was there any follow-up made after that …?

R: Yes, the information was relayed to the Wa Regional Health Directorate, specifically to one of the sub-districts there. They followed up by testing family members, and it turned out that the son of one of the family members tested positive.

I: Well, this question might have a few straightforward answers, but I’m curious about the location of your facility, its architectural design, and its administrative and managerial framework. Could these factors potentially expose your facility to future outbreaks? Are there any aspects of your facility—its position, location, status as a referral unit, size, or other factors—that might predispose it to outbreaks?

R: Geographically, the facility is easy to access. It’s situated on the main highway from .......*(name of city deleted)* to .......... *(name of city deleted)* . So, as long as one can reach the highway from their community, it’s easy to get to the facility. We’re located in .......... District *(name of district deleted)*, but the two neighbouring districts, ......... and .........*(name deleted)*, don’t have hospitals. They only have health centers. This makes us the only referral unit within our district and for the two neighbouring districts. As a result, we often receive patients from communities as far as .......... *(name of township deleted)* , which borders our district. Due to our accessibility, some community members come here for their healthcare needs.

One notable aspect of CHAG (Christian Health Association of Ghana) facilities, including .... ........... facility *(name deleted)* , is that we don’t operate with the attitude of “There’s no bed, so we won’t take you,” or “There’s no doctor.” That’s rarely the case at CHAG facilities. There’s a strong reliance on and trust in our facility. People know that when they come to .......*(name deleted)*, at least someone will attend to them.

Currently, our emergency department is housed in the same building as the Outpatient Department (OPD) and the administrative complex. So, OPD, emergency, and administrative functions all take place in the same building. This layout presents challenges. For instance, during COVID-19, the emergency cases and all the patients shared a common corridor that led to administrative offices, consulting rooms, records, and the lab. Structurally, this setup increases exposure risk, especially if we’re dealing with an airborne pathogen. Because of COVID-19, we moved the OPD waiting area outside, but the emergency department still has two wards on opposite sides of the corridor. Patients needing to use the bathroom, for instance, have to cross through this corridor, increasing exposure risk. So, our current OPD structure doesn’t allow for proper segregation and isolation when needed. We’re hoping to change this in the medium term, but, for now, these are some of the structural challenges we face.

I: Do you have ventilators or enough PPE and other necessary equipment?

R: We don’t have ventilators. We have oxygen concentrators but still rely on cylinders as well. In terms of PPE, we do have gloves and some supplies left over from COVID-19, so we are reasonably stocked on certain items. However, other items, like boots and aprons, are not well-stocked. We have some of these items but not in adequate quantities.

I: Let’s discuss your relationship with GHS (Ghana Health Service) and CHAG facilities. Specifically, what kind of arrangement enables GHS and your facility to work together on outbreaks?

R: Here, the relationship with GHS has been very cordial. Since I arrived, we’ve had two district directors of health, and both have made efforts to recognize our facility as the district hospital, treating it as such. The facility generates data we use for performance reviews, and we’ve reminded them that helping our facility indirectly benefits their data and district outlook, as we provide the majority of healthcare services in the district. The previous district director, for instance, would often walk in to discuss matters. On our end, the public health unit has worked closely with the district health directorate. It functions as part of the public health unit for the district, taking instructions and support from them.

Moreover, our public health needs, particularly in the ..... community *(name of village deleted)* , are primarily managed by our Community Health Nurses (CHNs) rather than the district’s public health officers. This arrangement allows our nurses to provide feedback directly to the district. It feels like we are working together as a single unit. Additionally, the district director provides supportive supervision and regular visits, and, in fact, there’s a team from the district here today working on breastfeeding initiatives.

I: It sounds like a smooth and effective relationship. Have there been any challenges working with the district health system?

R: Yes, it hasn’t always been easy. Although our working relationship is generally good, we do encounter conflicts at times. For example, they sometimes instruct our staff without informing us, which leads to duplication of efforts. When it comes to the distribution of assets, equipment, and consumables, we sometimes feel that the distribution is not proportional to our staffing levels or patient load. These issues can be frustrating, but overall, the relationship is better than some others. We make it work, although it has its challenges and isn’t as ideal as we would like.

I: I’m sure there are established policies that guide your expectations. Does the GHS have policies that define how your facility should interact with the district?

R: I’m not aware of any specific policy between GHS and CHAG or between the facility and the district. Based on my experience, a lot depends on the relationship between the district health director and the medical superintendent of the facility. If there’s mutual respect, things work well. However, I haven’t seen a policy that governs the relationship so that, even in the absence of animosity, there’s a framework to work together effectively.

I: Aside from the GHS, are there any other organizations you work with during outbreaks, like the Marburg outbreak? Did you collaborate with any organizations aside from GHS?

R: Yes, during the Marburg outbreak, the KCCR (Kumasi Centre for Collaborative Research) sent some representatives, and there were others from the GHS surveillance unit in Accra. The district health directorate coordinated the public education efforts with the district assembly. However, one challenge we encountered is that the district health management team (DHMT) does not include representatives from the hospital. This means there’s no doctor on the DHMT, so decisions are sometimes made without our input, and we later have to adjust to their plans. For the .......... community *(name of township deleted)*, our team handled the community education and engagement.

I: Have you ever needed to take an informal approach to get a service from any of these organizations or individuals during an emergency? How did you manage communication in such situations?

R: On a personal level, I often reach out informally to the district health director, the disease control officer, the district nutrition officer, and the district public health nurse. We frequently communicate by text and message even before official letters arrive. This informal communication helps us arrange meetings and relay information promptly. Sometimes, you receive a letter on the same day that action is required, so it helps when we already have a heads-up from these informal exchanges. Once the official letter arrives, we follow up formally, but this background communication is often essential.

I: Would you say that the informal approach is more effective or reliable than relying solely on formal emails or letters?

R: Yes, the informal approach is beneficial. While we eventually go through the formal channels, the informal method allows things to get sorted out faster and more efficiently than waiting for the official process.

I: When approaching external organizations like GHS or NCCE (National Commission for Civic Education), aside from human resources, what other resources do they provide to your facility?

R: In terms of the Marburg outbreak, we received some PPE from them through the district. Occasionally, the region provides PPE to all facilities within the region, both GHS and CHAG. Recently, we received an autoclave and a microscope. We sometimes get supplies if we push, but there’s no consistent provision. For example, last year, we received PPE on about three occasions, though it wasn’t much—perhaps a box of 100 gloves, one apron, and a few litres of sanitizing solution. We receive these items, sign for them, and thank them, even though they’re insufficient. At least we’re on their distribution list and get something, which isn’t the case for every facility.

I: We’re finished with our questions. Do you have any questions for us?

R: Yes, after we met with the director, I remember he mentioned that this interview was coming up. I just want to know the end goal of this interview and the purpose behind it regarding Marburg.

I: There will be publications from these interviews to highlight how facilities like yours manage outbreaks and the challenges faced. .........*(name of facility deleted)* name will remain anonymous, as will the names of all participants. The research will focus on CHAG facilities in the context of the outbreak, aiming to showcase how effectively we handle new outbreaks. Additionally, the article and analysis will be shared with other facilities to provide useful insights, similar to how we presented information during the summit. Those are the two main objectives that we’re aware of.

I: Thank you for your time.

SENIOR MEDICAL OFFICER (SMO)

|  | |
| --- | --- |
| PARTICIPANTS | R |
| INTERVIEWERS | I |

I: Sorry about that. Can you please tell us a little bit about yourself? We’d like to know your age, marital status, current designation, and how long you’ve worked in this particular district, and at your facility *(name deleted)*.

R: *Demographics deleted to protect participants’ identity*

I: Ok, thank you. As we mentioned earlier, the main discussion is about Marburg, specifically the Marburg cases that were detected in the facility. Could you please describe how it was detected? We aren’t focused on administrative details; we’re interested in what happened from the time the samples were taken until the disease was detected.

R: Alright, it’s been a while, but if I recall correctly, we recorded two cases in total.

I: Ok.

R: The first case came in—I think it was on the 27th of... I’ve forgotten the exact month.

I: That’s fine; you can proceed without the date.

R: When he came in, he was presenting with symptoms of diarrhea and vomiting. It seemed like a typical case of diarrhea with vomiting, so we initially treated him as if it was gastroenteritis. But on the second day, we realized he was now vomiting blood, and some of the stool was also turning bloody. So, we organized a blood transfusion, but within a few hours, he passed away. A sample was taken, probably about five days afterward, I think.

I: Five days after he died?

R: Yes, five days after he passed.

I: How long did it take for you to receive the results?

R: At that time, we weren’t suspecting Marburg. The Marburg suspicion only arose with the second case, which presented within that same week with similar symptoms of vomiting blood and bloody stools. That’s when we began to think something might be seriously wrong. So, we contacted our district director and decided to take a sample for further analysis.

I: Thank you. Regarding the sample collection...

R: The second case's sample was taken on the very first day he presented because, by then, we had seen two cases with similar presentations, and we wanted to understand what was happening.

I: Hello, doctor—can you hear me?

R: Yes, I can hear you.

I: Alright. So, please, with the first case, where the sample was taken five days after the patient’s death, where was the deceased's body kept between the time of death and sample collection?

R: Right. For the first case, because we were not initially suspecting Marburg, after the patient passed away, the relatives took the body. I believe the patient was from one of the towns in the Upper West region or possibly the Savannah region, so they moved the body there.

I: Ok.

R: Yes, by that time, they had moved the body to that area.

I: I see.

R: Yes, because at that time, we were not suspecting Marburg. It was only after the second case came in, showing similar symptoms, that we realized we had two patients with similar presentations and had already lost one. That prompted us to investigate further. For the second case, the sample was taken on the very day he presented to the emergency department.

I: Yes, so for the first case, you mentioned that the sample was taken five days after the person’s death. Was the body kept in a private morgue, or was it still in the facility?

R: Oh no, no. So, usually, when patients come in, we obtain blood samples as part of their initial workup since they are presented to the emergency. We already had a blood sample from the first case stored in our unit.

I: Ok, so you used the same blood sample; you didn’t collect a new one?

R: No, because by then, the patient had already passed away.

I: Understood, thank you.

I: When Marburg was detected in your facility, did it impact your daily medical practice and lifestyle?

R: Oh yes. During the initial stages, we were all quite scared because we know Marburg has a high mortality rate, and it was something new to us. Throughout my practice, I had never encountered a case like this; I had never even seen one before. I don’t know the total number of cases recorded afterward. Initially, we went through some psychological trauma because we had performed various procedures on these two patients. For example, we set up an IV line, and, with the first patient, we had to suture lacerations on the tongue when he was bleeding. We also inserted urethral catheters, set up intravenous lines, and performed other procedures. So, there was a lot of fear because you just don’t know if you’re going to contract the disease. For the first month or two, we experienced psychological trauma. We only felt relief after the test results came back negative. Even after that, we remained on high alert, and any case that presented with similar symptoms prompted us to run tests and rule out Marburg.

I: Understood. How long were you quarantined after being exposed to these patients?

R: We stayed in our rooms for about 3 to 4 weeks.

I: Ok.

R: Yes, if I remember correctly, about 3 to 4 weeks.

I: You mentioned that this experience affected you psychologically. So, were you afraid? What was the exact feeling you had?

R: Yes. The high mortality rate definitely weighed on us. Both of these patients passed away within just a few hours or days. We understood that if one contracts Marburg, there is a 50-50 chance of survival.

I: …

R: Exactly, we realized we could also die from it if we were infected.

Interviewer (I): So, did it affect your sleep? Did it affect your sleeping habits?

Respondent (R): Sleep? Yes, it affected my sleep because family and friends kept calling, and they were all wondering, at least hoping for some updates, even though I couldn’t give them much information about what was happening. We were worried about family and friends, you know? In case something happens to you, there are people who depend on you—parents, others who look up to you. So, yes, it was a difficult time.

I: Sure.

I: And, errr, was your eating habit or diet affected in any way?

R: Errr, okay, so regarding my eating habits…well, mmtcheeww, it didn’t affect me that much, but during that time, I modified my diet a bit. I wanted to include things that would help boost my immune system—mainly fruits and vegetables. You know, I felt that it would help my immune system, and we still had to be hopeful we’d get through it. So, yes, I focused more on fruits and vegetables. So, in terms of it affecting my diet, yeah, maybe in that sense, yes.

I: Okay, so throughout the quarantine period, while you were dealing with fear and anxiety, what helped you to cope? How did you manage all those mixed feelings?

R: Errr, how did I cope? Well, I mean, I don’t really know how I did it, but we all just held onto hope that the result would come out negative. We tried to continue with a sense of normalcy. Although we were home, we were just hopeful—all of us hoping the result would come out negative.

I: During that time, were you speaking often with your friends? And if so, were they encouraging you? Was their support helpful in keeping you positive?

R: Sorry?

I: I was asking if... Can you hear me?

R: Hello?

I: Hello?

R: Sorry, a call came through. Yes, I can hear you now.

I: I was asking if, during the quarantine period, you were speaking more often with your friends on the phone. The question is, in what way did your friends and family help you cope with the situation? Although you were in quarantine, how did your family and friends help you manage the anxiety?

R: Okay, so it was just, I mean, with me, per se, I couldn’t disclose to them that we had a case of Marburg in my facility. So, it was more of a normal conversation. It wasn’t like I informed my family or friends that we had gotten a case of Marburg. We just had our usual, everyday conversations. They didn’t know about the Marburg case in our facility—maybe they found out later on. But, at the early stages, when the initial case appeared, nobody knew it was from our facility.

I: Okay. But then, did you become more religious or more spiritual during that time?

R: Okay, well, I think I was just myself. I pray every day, so it was just a continuation of that.

I: I remember, in my case, when COVID broke out and someone at my workplace had COVID. Knowing I had interacted with the person, I was scared to death! I prayed, like, ten times a day! Hahahahahaha!

R: Wow, wow! I didn’t know! For me, it was just my normal daily prayers.

I: That’s fine.

R: Yeah.

I: But then, what new measures did your facility put in place in response to the Marburg outbreak?

R: Yes, so during that time, all the prescribers, especially the emergency nurses and OPD nurses, we had a meeting. We discussed the case definition—how to recognize it, when to suspect it, and so on. We wanted to be able to identify it, so we could do further examinations and questioning. If lab tests were needed, we would inform the district director, disease control, and all that. So, on the clinical level, we discussed the case definition and what symptoms to look out for. But on the management level, we had a major challenge because we didn’t have an isolation center.

I: Hmmmm.

R: In bigger health centers, you’ll find isolation centers and facilities for disease control, but here, we didn’t have one, which was a big challenge. We had to use one of the emergency rooms that normally accommodates two or three patients. We had to clear out all the patients from there and designate that room solely for suspected cases of Marburg or any contagious diseases. But even that was still a challenge because that room doesn’t have a washroom. If someone is isolated there, they would still mingle with the other patients in the emergency ward, which made things quite challenging. Clinically, we were looking out for symptoms like fever, muscle pain, vomiting, bleeding, or vomiting of blood—anything to help us pick it up early.

I: Okay. But did any of the measures put in place affect you in any way, either positively or negatively?

R: Ohh, errm, well, the measures were supposed to help us respond quickly in case another Marburg case appeared, so we could identify it early. So, yeah, the measures were there to make sure we wouldn’t miss it like we did with the first one.

I: Okay. But when you were exposed and in quarantine, did you receive any support from the institution?

R: Errmm, yes. When we were under quarantine, the management provided some food items for us.

I: Okay.

R: Hello?

I: Yes, I can hear you.

R: Yeah, yeah. They provided us with food items; we were supported by the hospital management.

I: How did the facility monitor those on quarantine to ensure they didn’t go out? I guess you were advised not to go out because you were in quarantine, but there may have been emergency needs that needed to be met. How did they monitor to ensure people didn’t leave quarantine?

R: Okay, so, errmm, we actually classified the exposure levels as low, moderate, and high risk. Those of us who performed procedures, like suturing, were classified as high-risk. Most of us who were in that category were prescribers.

I: Okay.

R: And, as prescribers, we stayed on the hospital premises; we had an apartment here.

I: Okay.

R: So, it was easy for them to monitor our movement because we were all in the same block. If anyone tried to leave, others would notice and say, "Hey, you’re supposed to be in quarantine, where are you going?" That part was, I think, manageable. The challenge was more with those at low risk—like patients who only administered basic tasks, like checking vitals. They didn’t have physical contact with body fluids or a blood splash, so the risk of contracting the virus was very low for them. However, they lived in town, so monitoring was a bit challenging. The district, however, put measures in place. They had staff monitoring those who lived in town, and they also monitored the relatives of the patients involved in these cases. The district director made sure there were people checking on them, especially the relatives of the two cases.

I: Okay, okay. How effective was the contact tracing after detecting the cases?

R: The contact tracing? Errm, could you say that again?

I: How would you rate the effectiveness of the contact tracing?

R: Okay. The contact tracing—I would even rate it, if I’m not exaggerating, like 99% or 100%.

I: Okay.

R: The people assigned went to the neighborhoods of these two cases to check on their relatives almost every day. Throughout the three-week incubation period, they checked regularly, asking, "Are you developing any symptoms?" and things like that. It was very, very effective; it really helped. So, yes, the contact tracing was excellent.

I: And, with your experience, were there any institutional policies or programs during that period aimed at managing the outbreak that you felt could have been handled better? Anything you weren’t comfortable with?

R: Did you say, in my experience?

I: Yes, based on your experience during that period. Were there any programs or policies initiated by the facility to manage the outbreak that you felt could have been improved?

R: Yes. The first thing I’d mention is that not having an isolation center was a big, big drawback. The room we designated to manage the case was even located in the OPD area, right near the administration center—the main part of the hospital. So, not having an isolation center was a major challenge.

I: Do you have a morgue?

R: Sorry?

I: You have a morgue or a mortuary at the facility?

R: I have a…

I: Does the facility have a morgue or a mortuary?

R: A morgue? No, currently, the facility doesn’t have a morgue.

I: Please go ahead. You were explaining other areas that could be improved.

R: And so, I think that was the major challenge…yes, that was the challenge, because apart from that— I mean, the samples being taken, us getting the results and all that—I think it was okay. I wouldn’t say there was any delay or anything like that, apart from the fact that, okay, on a clinical level, we missed the first case. But, I mean, for people to be able to identify that this is Marburg… that would be very, yeah…

I: When we look at the decontamination process that took place after the case was detected, how would you describe it in your estimation?

R: Yes, yes, so once we received the result that the second case was positive for Marburg, we had to discharge all the patients, even some critically ill ones. In fact, we referred some of them to nearby facilities so that we could make the ward safe—both the general side and maternity ward, and even the theatre. Everything was put on hold. We then called in the disease control team to spray these areas and disinfect thoroughly. So, yes, we were able to disinfect most of the wards, rooms, the theatre, maternity ward, and general ward. Everywhere— we had to send everybody home. For the critically ill patients, we transferred them to our nearby facility, which is Bekwai Hospital, so they could continue managing those cases.

I: Okay. After the second case, you were now aware of what you were dealing with. How did it affect the evacuation process?

R: How did it affect the evacuation process?

I: In other words, how did the evacuation go? Were you able to gather the equipment, PPE, and other essential materials needed for the process? How fast did it go? Were you able to get an ambulance on time? Were there any challenges in handling this emergency, or would you say the process was swift and efficient?

R: In terms of the response after we had these cases… yeah, yeah, after these cases, they supplied us with some of the PPEs since we had been facing challenges in that area. The district director supplied us with some PPEs, especially to help with the emergency and OPD cases. We even had the president and vice president of the Church institution *(name deleted)* come in, as well as representatives from CHAG, who held a meeting with us to understand the situation and see where they could assist us with the challenges we were facing. So, yes, the response was good.

I: Okay, okay. And you mentioned earlier that after the cases were detected, you called in the disease control team to evacuate the wards and remove the patients. How did you communicate to the patients about why this was happening?

R: The response was good.

I: And, by extension, how was this information communicated to other clients who visit the hospital? How was the message conveyed to both those in the ward and the general client base?

R: Okay, okay, so what we did was not an abrupt announcement. Because of the stigma and other factors related to our location, we couldn’t just tell everyone or publicly announce, “We have Marburg cases.” So, because of the stigma, we weren’t accepting new cases. For the patients who were already in the ward and were stable, we quickly discharged them. For those we thought might have complications or would need to stay longer, we referred them to other facilities. It took several days for us to gradually clear all the patients out.

I: In an ideal situation, would you have done anything differently to improve the facility’s human resource capacity to handle such an emergency? Is there anything lacking in terms of human resources? If you had the chance to improve, what would you add to the existing setup?

R: That would boil down to the need for an isolation center, because with an isolation center, you’d have specialized staff, such as nurses, anesthetists, emergency care providers, emergency physicians, or infectious disease specialists to manage the cases. So, having an isolation center comes with this whole package. If anything, that’s what I would say needs to be added.

I: And just related to that question, if there’s a new situation or outbreak, does the facility usually assemble staff for specific training to manage it? During the Marburg outbreak, what kind of training did the management organize, and how did it go?

R: During that time, our focus was on the emergency and OPD nurses. We held a clinical meeting—yes, a clinical meeting—where we went through what Marburg is, how to identify it, what symptoms to look out for, and who to notify. We instructed that if a patient comes to the OPD with certain complaints, like vomiting blood or diarrhea, they should notify the prescribers or doctors immediately and triage the person. We emphasized monitoring patients coming from areas where the disease had been reported, such as Asokwa. So, we held a small clinical meeting with most OPD and emergency nurses, along with the prescribers, on how to manage the cases.

I: We’re almost done and won’t take much more of your time. Regarding the facility management system, are you aware of any standard operating procedures (SOPs) for handling emergencies or outbreaks like this? Is there a policy within the facility for managing such situations?

R: Sorry, could you repeat the question?

I: Is there a standard operating procedure within the facility for managing emergencies, such as Marburg or similar outbreaks, that would serve as a guide for the staff?

R: So, generally, we do have an emergency unit to handle all emergency cases. The staff and nurses in the emergency unit participate in workshops and educational sessions, and we hold clinical meetings to discuss various medical and emergency conditions.

I: Okay. But is there a specific policy or standing plan that, for example, was used during COVID-19 or for Marburg? A set protocol that staff could refer to, stating exactly what needs to be done in these situations?

R: Yes, we have that. For COVID, we implemented protocols, and even for Marburg, we followed a similar approach during our clinical activities.

I: Okay. We’re almost done with our final set of questions. You previously mentioned the support you received from the district directorate. Aside from the PPE they provided, what other types of assistance did you receive from them to manage the outbreak?

R: Oh, excuse me—a call came through.

I: No problem. I was asking, aside from the PPEs provided by the district directorate, what other assistance did they offer to manage the outbreak?

R: Right, so they handled tasks like blood sampling and follow-up. The district disease unit took the blood samples and managed all the testing. After we confirmed Marburg, there was an incident where a blood sample splashed on one of our student nurses who was doing his clinical internship. About a week or two after that, he was rushed to the emergency ward due to bleeding, and we became very concerned that he might have contracted the virus. During that time, the disease control unit was extremely helpful, providing support for the nurse throughout the entire process. They were working hand-in-hand with us, visiting daily. The district director was also very involved and supportive.

I: Given that, how would you describe the relationship between your facility and the district health directorate?

R: Oh, it’s like a family relationship. Even before Marburg, the director has been coming here, even attending clinical meetings. If there’s neonatal mortality, for example, he still comes around. So, we are like a family—there’s no bad blood between us.

I: But have you faced any challenges in working with the district health system? Any issues, for example, in responding to other disease outbreaks like Marburg or COVID-19?

R: (Chuckles) No, we haven’t had any challenges. Their responses are always prompt. Once we inform them, they show up right away.

I: You mentioned CHAG as one of the agencies that visited after the case was detected. What assistance did they provide, aside from the visit?

R: Yes, CHAG and the Church *(name deleted)* came by.

I: Okay, so aside from their visit, did they provide any other assistance?

R: We had a meeting with them to explain the situation—what had happened, the steps we’d taken so far, the issues we were facing, and what they could do to assist the facility.

I: Okay, okay.

R: I remember, at that time, I presented to them that our main challenge was the lack of an isolation center, especially because it was still early in the outbreak, and we were unsure where to keep new Marburg cases if they arose.

I: Aside from these institutions, we have information that the first case was referred through your facility. There was some engagement between your facility and ....... Hospital, correct *(name deleted)*? Could you elaborate on any collaboration you had with them since the case passed through their system before arriving at your facility?

R: Which facility?

I: We understand the first patient had gone to ......... Hospital *(name deleted)* a few days before he arrived at your facility.

R: Okay, okay. So even for the first case, he was working at Bogoso. He was involved in "galamsey" or small-scale mining at Bogoso, and that was where everything started. So, his friends told him to come around. I think it was around the same time that things weren't going well for him with the galamsey work. So they advised him to come over to Kusa—yes, Kusa, which is on the outskirts of ........*(name of town deleted)*. When he came here, he told them he hadn't been feeling well for a while. They mentioned there was a health center nearby, so they took him to ............*(name of town deleted)*. After his visit to ..............*(name of town deleted)*, he eventually ended up here.

I: Okay, okay.

R: (Laughs) So, they referred him here because the health center at ..........*(name of town deleted)* has some limitations when it comes to administering IVs, certain medications, and certain types of care.

I: Okay, so when his case was detected, and you informed the relevant parties, was there any collaborative work between the two facilities?

R: Yes, yes, yes. So, all those processes were managed by the Disease Control Unit. All the nurses who came into contact with him were part of the contact tracing efforts.

I: Okay, alright. So finally, in terms of contacting any of these facilities or institutions—whether the district directorate, CHAG, or any other institutions—was there any involvement from civil society organizations or any community groups engaged in this?

R: Yes, yes. The Ghana Health Service involved us—the GHS brought us in. Oh yes, within just a few days. So, essentially, the Ghana Health Service, and there was also another organization from Accra. We had a Zoom meeting with them, along with the medical director of our facility. Almost all the high-level authorities were involved and engaged with us.

I: For the final question—when you were reaching out to these organizations, were there times you needed to use informal methods? Sometimes informal processes can produce faster and more effective results than the formal route, like sending emails, writing on letterheads, and going through all the official protocols. Were there instances where using an informal approach proved to be more effective?

R: Yes, there were some Zoom meetings, especially organized by the Ghana Health Service and the regional headquarters. So, those meetings helped facilitate things because they could ask questions directly, and we could respond right away. So yes, I think that facilitated the process quite a bit.

I: Dr. Ebenezer Arthur, thank you very, very much. We have gone through all of our questions, but we want to check if there’s any additional information you’d like to share with us. Is there anything else you planned to tell us about the Marburg case that we haven’t covered?

R: I think we’ve touched on almost everything.

I: If you have any questions for us, we’re here to answer them. And if we don’t have the answers now, we’ll get back to you later with them.

R: Hmm, no, I don’t think so.

I: In that case, we are very grateful for your time. Thank you for spending nearly an hour with us. Please have a pleasant day.

R: Alright.

I: Enjoy your week.

R: You too.

I: Bye.

BIOMEDICAL SCIENTIST (BS)

|  | |
| --- | --- |
| PARTICIPANTS | R |
| INTERVIEWERS | I |

I: This process is primarily for research purposes, so all the information we collect will be kept completely confidential. Your name and any specific identifying details won’t be attached to the data, so no one will be able to tell that you’re the one giving this information. You’re free to speak openly with us.

R: That’s fine. I understand.

I: Alright. We’d like to start by getting some basic information about you—your age, marital status, and so on.

R: *Demographics deleted to protect participants’ identity*

I: You mentioned that you’re a biomedical scientist, correct?

R: Yes, that’s correct.

I: And what is your current grade or designation?

R: I’m a Senior Biomedical Scientist.

I: Alright. Thank you for that. You can turn off your video if that makes you feel more comfortable.

I: Hopefully, that will help the internet connection remain stable, right?

R: Oh, okay. Thank you.

I: So, when Marburg was detected in your facility, we understand that some people were quarantined. Were you one of the people asked to quarantine?

R: No, I wasn’t. After discussions with my medical director, it was determined that my contact with the sample was minimal, so I wasn’t required to go into quarantine.

I: Alright. But what measures were put in place at the facility to prevent the spread of the virus during that period? What specific steps were taken in response to the outbreak?

R: Yes, so the first measure was to quarantine any staff who had direct contact with the patient. I also doubled as the IPC (Infection Prevention and Control) focal person for the facility. Within about three days of getting the news that the samples we sent had tested positive for Marburg, we quickly organized a training session for all the staff on standard and transmission-based precautions under IPC. We didn’t have specific training tools for Marburg, so we focused on standard and transmission-based precautions.

I: Okay. Can you provide specifics on the precautions and measures that were covered during the training?

R: Certainly. We covered all 12 standard precautions, including hand hygiene, respiratory hygiene, and everything else under standard precautions. Since we knew Marburg was transmitted through contact, we placed extra emphasis on contact and transmission precautions. We went back to some of the measures we used during COVID, like spacing out patients for social distancing, especially in the OPD (Outpatient Department) and vital signs area.

I: Mr. Bentum, you mentioned the 12 standard precautions. We’re very interested in knowing exactly how each was carried out. If you need a moment to refer to your notes or recall the details, please feel free to do so.

R: Okay, let me check my notes. It was mostly a theory session with some practical demonstrations. For instance, for hand hygiene, we went through the practical aspects of washing hands thoroughly with soap under running water and using alcohol-based hand sanitizer. Let me take a minute to refer to my notes for more specifics.

I: That’s perfectly fine. Take your time.

I: Hello, Mr. Bentum?

R: Sorry, my network went out briefly. Can I continue now?

I: Yes, of course. Welcome back.

I: That’s fine. Please go ahead.

R: So, we covered hand hygiene thoroughly. We also reviewed respiratory hygiene, which was particularly relevant because we’d recently come out of COVID. We covered the collection of clinical specimens, occupational health and safety, safe injection practices, aseptic techniques, and healthcare waste management—especially for our orderlies. For the first two cases of Marburg, our orderlies weren’t initially aware they’d be dealing with it, so they just went in as they would for any routine cleaning. However, after we learned it was Marburg, we trained them on how to handle waste from the isolation room specifically. We also covered environmental cleaning, processing of used instruments, proper patient placement, and the use of PPE (Personal Protective Equipment), especially for the clinical staff who might handle cases directly.

I: From your experience, did you have enough equipment, like PPE, to properly train the staff and manage the outbreak?

R: Yes, so the training was done in two sessions. The first session covered standard precautions, and the second focused specifically on the emergency unit staff once they returned from quarantine. We had made a request to the District Health Directorate for additional PPE, so we had enough at that time to train them adequately.

I: And how did this experience impact you personally, in terms of your relationship with colleagues and others?

R: Personally, it was quite challenging. I wasn’t really expecting something like this, especially since we were barely out of the COVID crisis. When I first handled the sample, I knew they suspected some kind of viral hemorrhagic fever, but I initially thought of Ebola because of its presence in the region. When I later learned it was Marburg, it hit me hard. I realized this was something serious. The two cases didn’t know each other, yet both contracted the virus. I actually lived between the two hotspots, so I was extra cautious in everything I did. With my colleagues, it strengthened our sense of support. I remembered how I felt when I was quarantined during COVID, so I tried to check in on the colleagues who were quarantined, calling them to offer support. When their test results came back negative, we were all relieved and happy to have them back.

I: Was there a counseling program in place at the facility to assist those who were quarantined or anyone who might have needed mental health support?

R: I can’t speak for everyone who went into quarantine, but for myself and some colleagues, the facility has a mental health unit where we could walk in and talk if needed. Personally, I didn’t feel like I needed formal counseling at the time. I did talk with my nurse manager about a few things, and that helped. But I can’t say if others felt the same way or if they used the mental health unit.

I: For those who were quarantined, what mechanisms were in place to ensure they stayed at home? I assume it was a self-quarantine situation?

R: Yes, it was self-quarantine. I don’t know if there were specific measures in place to enforce it, though. I only kept in touch with a few of them over the phone to check on their well-being, but I can’t say for certain that there was a way to monitor whether they stayed home the entire time.

I: Understood. On a broader level, were there any standard operating procedures or policies in place at the facility that you relied on whenever there was an outbreak or emergency situation?

R: Yes, we do have such procedures in place. COVID really pushed us to improve in that area. Previously, our triage system wasn’t very well organized, but COVID made us start implementing better triage measures. Our isolation room still isn’t quite where we’d like it to be from an IPC perspective, but we’re continuously working on it and seeking support to improve it. COVID, followed by Marburg, has definitely pushed the facility to start putting some of these measures in place to better handle any future outbreaks.

I: Alright. So, during the outbreak response period, were there any institutional measures or new regulations put in place that you felt uncomfortable with? Maybe something you thought could have been handled differently or improved upon?

R: From the facility’s perspective, no, there weren’t really any major discomforts. However, on a broader systemic level, I remember that the sample results took about a week to come back. During that waiting period, there was a lot of uncertainty; you didn’t know what was happening or what to expect. So, from a system-wide perspective, the delay made it a bit challenging, but at the facility itself, I didn’t have any specific issues with the measures in place.

I: Thank you for that. Now, regarding the evacuation of the deceased—do you have a morgue in the facility?

R: No, we don’t.

I: How long did it take for the deceased to be evacuated, and how did that impact your procedures during the period?

R: If I remember correctly, the first case was a Muslim patient, so we had some challenges coordinating with the relatives on how to proceed. However, by the time the second case occurred, we had already informed the District Health Directorate about the situation. Fortunately, they were available to assist us with the second case. Following IPC guidelines, I believe we managed the second case appropriately.

I: You mentioned the cleaning staff earlier. For clarity, can you describe what they usually wear and the equipment they use? Does it differ during emergencies compared to normal times? It sounded like they might not have been fully prepared at first.

R: Yes, in routine situations, the cleaning staff usually wear basic gloves, aprons, and occasionally face masks when dealing with high-risk areas. However, in the case of an outbreak like Marburg, they needed to wear full PPE, including gowns, face shields, masks, and double gloves. Initially, they weren’t fully aware of the risk and went in with just routine protective gear. After we realized the seriousness of the situation, we upgraded their equipment and gave them specific training on handling high-risk cleaning, especially for waste management from isolation areas. For IPC purposes, our trainings throughout the year usually serve as refresher courses to remind everyone of the protocols. Our orderlies have scrubs they wear at work; they don’t wear their personal clothing while cleaning. After work, they change back into their clothes to go home. I made that comment earlier because the equipment they use varies by area. For instance, what they use to clean the lab differs from what they use in the emergency unit or administrative offices. The emergency unit has its own cleaning equipment, and our makeshift isolation room requires specific PPE and equipment. Initially, they were just going in with standard equipment without knowing there was a Marburg case. After we confirmed it, they were required to use additional protective gear.

I: That makes sense. Thank you for clarifying. Regarding communication, how was information shared with the staff and patients during the outbreak? Were there specific steps taken to control the information flow, especially since we understand the OPD and isolation room are quite close?

R: From my perspective as both the head of the lab and the IPC focal person, information was shared first with me and…

I: Okay.

R: So, once I was informed, I had to relay the information to the staff in the lab because, yes, the lab had sent some samples for viral hemorrhagic fever, and we were waiting to get feedback. So, once that confirmation came in, I informed the lab staff about what had happened. Then my next line of communication was the IPC team. I would assume that from the medical director's point of view, he informed various management members, the mid-management members, and the heads of different departments. From there, the information would go through the ranks. Now, our isolation area was labeled—there were notices at the front indicating that it was an isolation area, with instructions on IPC practices, so people didn’t just walk in. Additionally, in the OPD, they conducted health education every morning. After all the staff had been informed that we had recorded two Marburg cases, the health information team took it up and started discussing it at the OPD to keep everyone informed.

I: Eeeerrrmm, hold on a second, please.

R: Could you please repeat?

I: He hasn’t actually asked the question yet. But, looking at your facility’s location and the structures you have in place, do you think there’s a heightened risk for disease outbreaks?

R: Eeerrrmm, I wouldn’t really want to answer that because I may have personal reservations about it. But from the facility's standpoint, I don’t think my position is the right one to answer that question. That’s just how I feel.

I: Basically, we’re just trying to understand the architectural design, proximity, and layout—like you mentioned earlier about the OPD being close to the isolation unit. Also, considering that your facility might act as a referral center, how could the design and location of your facility potentially influence future outbreaks? It’s not a policy question; we’re just trying to get a mental picture of your facility's setup and understand if it could account for frequent outbreaks.

R: Alright. So, the facility was initially a health center and later transitioned into a hospital. Within the district, the only other facilities are health centers, so we act as the main referral center here. In terms of our architectural design, I wouldn’t say we’re well-prepared for outbreaks. For instance, the isolation room is situated such that you pass through the OPD to get to it—there isn’t a second door for an alternate entry or exit. From an IPC perspective, no, we’re not adequately prepared for outbreaks.

I: Alright, alright. And what kind of relationship or collaboration does your facility have with Ghana Health Service, CHAG, or any other agencies?

R: I think my medical director would be able to provide more details on that. But I know that we informed the District Health Directorate when we picked the samples, and they were the ones who sent them to Noguchi. When the results came back, they reached the district level first, and they informed us. Any PPE we needed was also provided through the District Health Directorate, so we had good collaboration with them.

I: Okay. So, aside from PPEs, did you receive any other assistance from the GHS?

R: From my position, I may not know the full extent. I was mainly involved in securing PPEs and sending the samples. But beyond that, I’m not sure if there were any additional resources we received.

I: Alright. Based on your experience and observations, do you think you had enough support from the GHS to manage an outbreak like this?

R: Yes, I think we did.

I: Okay, and regarding the facility from which the first case was referred, what kind of collaborative system existed between your facility and theirs, especially after you detected the case?

R: I think we have a strong relationship with them. In the lab, for instance, we coordinate very well with their lab staff. We have a good rapport, particularly in terms of logistics, trainings, and other matters. Since we’re within the same district, we work closely together. I believe that this level of collaboration is mirrored in the nursing and prescriber levels as well, though I’m more familiar with the lab side of things.

I: Okay. So, when it came to contact tracing and other activities following the detection, how did that process go?

R: Contact tracing was carried out in collaboration with the District Health Directorate. I remember that the District Health Director, along with the public health nurse and the disease control officer at the district level, led the efforts. We followed them as they collected samples and identified contacts.

I: For the first family, who you mentioned were Muslims, the deceased was taken by the family. How did that impact contact tracing efforts, especially getting into their home or community?

R: Contact tracing for the first family was challenging. I remember when we went to collect samples from the community, it was difficult to reach them because I believe they had left the area and gone back to their hometown. So, from the information I gathered, it was quite difficult to trace them.

I: In terms of community relations, specifically the community where your facility is located, how was the collaboration between the facility and the traditional leaders?

R: That’s a bit difficult for me to answer. I don’t really have much information on that aspect, so I’m not sure what took place in that regard.

I: Okay. Aside from the Ghana Health Service, did you have any collaboration with CHAG?

R: I’m not aware of any collaboration with CHAG at my level. If there was, it might have happened at the administrative level, but I didn’t see any direct involvement in my position within the lab.

I: I see. And aside from state institutions, did you notice any involvement or assistance from non-state agencies?

R: Yes, I think there was some involvement. When the outbreak happened, there were a series of meetings with different stakeholders, including representatives from WHO. In January, for instance, there was a training session led by the senior IPC in collaboration with some NGOs that came to support the district.

I: Now that we’ve seen partnerships between your facility and both state and non-state institutions, can you briefly describe any existing policies that govern these collaborations? Are there any policies specifically for handling outbreaks?

R: As far as I know, I’m not aware of any specific policies outlining such collaborations. If they exist, I haven’t been privy to that information.

I: Alright. Thank you. Regarding collaboration, were there any forms of assistance you were hoping for but didn’t receive?

R: Eeerrrmmm, well, probably infrastructure support. For example, our isolation room is not up to the standard we would like. If we could receive assistance to improve the isolation facilities, that would be helpful. COVID and now Marburg have shown us the importance of these facilities. Infrastructure for the lab would also be appreciated. When a team came for research purposes, they were surprised that we could pick samples, store them, and transport them from such a small lab. They were genuinely surprised to learn that we managed to do that from this setup.

I: Is the lab that small?

R: Yes, it’s quite small. We’re hoping for an expansion that would provide us with the necessary logistics and equipment to handle cases more efficiently. In terms of movement, we don’t have the standard space required in a lab.

I: So, at any given time, can you only have one person in there?

R: Yes. For sample collection, only one lab staff member can be in the area with the patient at a time. It’s primarily about maintaining privacy, but we also lack the space to accommodate multiple staff members taking samples simultaneously. Although we might have four or five staff on duty during morning shifts, we have to be careful not to crowd the lab, as it limits movement.

I: But do you have enough staff overall?

R: Eeerrrrmm, yes. Management is working on it. We submitted our human resource needs, and they’re actively addressing them. We recently had one new person join the team in the last three months, so there has been progress.

I: You hesitated a bit. Is there anything specific you wanted to share?

R: Well, we don’t have the ideal numbers we would like, but we understand that management is working on it. Progress is being made, and, as I mentioned, we’ve had one new team member recently, so things are moving forward.

I: Alright. Final question—when you need to call the directorate or another agency for assistance, how does that communication work? Is there a formal or informal process?

R: Eeerrmmm, I wouldn’t really know the exact details of how it’s done. My role is to communicate with the medical director, and from there, the communication continues up the chain. I can’t bypass the medical director to contact the District Health Directorate, so I can’t say exactly how it proceeds from there.

I: That’s fine. Thank you very much. Is there anything else you’d like to add or any topic you think we missed?

R: Eeerrmm, no, not really. I just hope my responses were helpful. Some questions, especially those about collaborations and policies, were beyond my level, so I can’t fully answer those. But I tried my best.

I: You’ve been very helpful, and we’re grateful. Is there anything you’d like to ask us?

R: No, not really. But the lab unit and the facility would certainly appreciate any feedback or outcomes from this study.

I: Absolutely. One of our main objectives is to share the results with participating facilities.

R: We would be very grateful for that.

I: Thank you very much, sir. Have a pleasant day.

R: Thank you. You’re welcome.

I: Goodbye.

SENIOR HEALTH SERVICE ADMINISTRATOR (SHSA)

|  | |
| --- | --- |
| PARTICIPANTS | R |
| INTERVIEWERS | I |

*Demographics deleted to protect participants’ identity*

I: How was your experience with the detection of Marburg disease in terms of the administrative processes? When the case was reported and it was detected that this was unusual, what actions were taken by the hospital until the results came in and confirmed that it was Marburg?

Respondent (R): It happened on a day when I saw our district director around, looking busy and quite disturbed. So, I asked the director, “Are we safe?” He informed me that he would meet with me shortly. I then saw the director interacting with some of the clinical staff, and later he came to my office to brief me. He told me that he had received a call from one of the doctors who suspected a public health case here and wanted them to come and check it out. Prior to that, we had a collaboration with him, although there’s a new person in charge now. Back in 2019, ..........*(name of facility deleted)* became like a second facility to him, so when we had new clinical staff, such as prescribers, he came to take them through disease control surveillance. I believe that training helped us a lot.

The director explained that he had received a report of one case from the .......... area *(name of township deleted)*, which isn’t in our district, and another case from our district. Both cases had the same symptoms and signs, and the first one had unfortunately died. This made them alert, so they took samples and sent them to the directorate. We are a referral center, and since the first case had died, the staff at the district were alarmed. We quickly organized to prevent any spread and to alleviate any fear or unnecessary anxiety among our staff.

The medical team and administration called a short meeting with staff who had been involved in attending to the cases, and we identified those who had direct contact with the patients. We continued tracing from one staff member to another and identified around 21 individuals. I apologize; I can’t remember the exact number. After identifying the contacts, we asked them to self-isolate for the incubation period and undergo monitoring. We sent them home and management organized provisions for them to reduce the need to go out. We assigned a team member to check on them regularly to see how they were doing.

During this time, we also revived our Infection Prevention and Control (IPC) team and our emergency preparedness team. We don’t have much space here, so we designated one room for isolation. The IPC team inspected the area, performed the necessary decontamination, and conducted refresher training on protocols for the staff. We also held a staff meeting to inform everyone about the situation and the necessary steps we were taking.

Asking staff to go into self-isolation was challenging for our HR team because, at the time, we had only two prescribers, and both were involved in the case. Our Physician Assistants (PAs) were also involved, so our OPD (Outpatient Department) was at risk of being shut down. The Medical Director (MD) reached out to the regional director, who then authorized us to write to his office so they could spare us some staff for a short period. Fortunately, our diocesan director provided us with a PA for locum, and another PA who had completed his national service here offered to assist. This allowed us to keep the OPD functional. Additionally, a doctor we sponsored to train in China had returned and was waiting for posting, so he helped support the inpatients during that period. This solved our HR challenge.

We received a visit from the Church institution which owns our facility *(name deleted)* during this period, and we informed them of our need for PPEs. CHAG (Christian Health Association of Ghana) and the district director donated PPEs, which greatly supported our staff. We also had follow-up visits from CHAG and Church institution *(name deleted)* directors the following week.

I: Regarding the materials you needed to contain the spread, do you think you had enough to manage it?

R: No.

I: Which resources were lacking?

R: I would focus on the isolation center. When it comes to controlling the spread of disease, we don’t have enough rooms. We worried about how we would manage if more cases emerged. The isolation center is located right on the corridor of the OPD, close to the emergency area, which is also where the administration is located. While this setup worked in the interim, it’s not ideal. We don’t have an ICU or ventilators—only the basic resources needed to manage cases. That’s all I have to say.

I: Do you have a morgue?

R: No, we don’t.

I: How did the lack of a morgue affect the evacuation of deceased individuals?

R: Since we don’t have a morgue, we rely on a private facility at Asokwa Junction. When cases expired, we wanted to avoid creating tension, so family members came for the bodies. The district director tracked these cases as they were public health concerns and managed them for us. One of the deceased was sent up north, and contact tracing became challenging because of that situation. Once we confirmed it was a Marburg case, we had to trace where the bodies had been sent, which created a host of issues. ..........*(name of facility deleted)*  is small, but recently, the board has approved plans to start a mother and child care center and a morgue.

I: Regarding the case that was taken up north, do you know how long it took for the body to leave the facility?

R: No, I don’t have information on that.

I: You mentioned how information was communicated among staff. You were briefed, and training was organized. Was any information shared with patients, especially in the ward where the first case was identified? How did they understand the term “Marburg”?

R: We hold morning health education sessions led by our public health team. The director gave us some banners and posters, and our trained staff also shared information with patients during OPD hours. We emphasized the need to wear masks and essentially revived all the COVID-19 protocols.

I: In terms of security, how did you control the movement of people to ensure that protocols were observed?

R: We set up a washing area at the security checkpoint and provided masks. If anyone arrived without a mask, security would give them one before allowing them to enter. We also had staff routinely check the waiting area to ensure everyone was wearing masks. Thankfully, many of our key staff members, including security and orderlies, have a strong sense of ownership here since they were part of the hospital’s foundation. They understand our protocols and limit access to restricted areas. Even community members cooperated with our protocols, so there was no panic.

I: Maybe they didn’t fully understand Marburg?

Interviewer (I): Can you describe how the news of the outbreak was received here compared to outside of Dumpoasi?

Respondent (R): No, that’s not it. How we received the news outside Dumpoasi wasn’t what was really happening here. Personally, I had a call from a friend, and the person—speaking in Twi—mentioned she heard there’s a disease here and people are dying, hyping the condition. I didn’t want to disclose that it was my facility. It wasn’t like that at all. Our district director, thank God, is a public health person. So, in this case, he personally went to the market and spoke to the market women, just like he did during COVID-19. He assured them that, just as we handled COVID-19, we would handle the Marburg outbreak in the same way. So there wasn’t much panic among those here, although they had heard about the disease. The confidence was there because they were assured, but outsiders were panicking more than those inside.

I: It sounds like your facility had a standing plan for managing outbreaks.

R: The readiness is there, but the equipment is not.

I: So, there isn’t a plan or strategy that you can rely on in case there’s an outbreak?

R: As I said, the isolation room is the only dedicated area we have for that purpose, and it has only two beds.

I: Are there any policies or standard operating procedures that you always adopt when there’s an outbreak like this?

R: Yes, there’s a protocol for outbreaks.

I: And you usually use that every time an outbreak occurs?

R: Yes. Even to prepare ourselves for the outbreak, when we first hear of it... Today, I had... There’s a Lassa virus alert, so the team will be preparing to start being on guard.

I: Now, we want to talk about you personally. How did the entire situation affect you? Did it impact your response to work? Did you have to make any personal lifestyle changes? How did the whole experience affect you?

R: I’m a public health person; I studied public health at Catholic University, so I found it interesting to learn more about the case and how it was being managed. I saw it as a learning point. Personally, it became an all-hands-on-deck situation. It wasn’t like this is for clinical or for administration. No—I was in contact with the nurse manager, who was monitoring the IPC (Infection Prevention and Control) team. I joined him to learn more and build on my public health experience, which I hadn’t actively used for some time. So, there weren’t many changes; I just took it as an opportunity to learn.

I: How did you collaborate with the facility that referred the first case to you? How did you manage the process of getting information across, and what kind of assistance did you give each other?

R: They have a WhatsApp platform for referrals to the facility. So, if they are referring a case, usually the one referring will just send a message saying, “I’m referring such and such a case,” or they’ll call and inform a colleague that they are bringing this case here. And as I mentioned, this facility is like a secondary hospital for the district director, so that collaboration exists. You see him here almost every Monday for updates. He was also a board member, so he was taking information here and relaying it to the directorate and the facility located on the directorate’s premises.

I: So, are you referring to the district director?

R: Yes, the district director and his team.

I: It seems you have a very good relationship with him.

R: Yes. When it comes to the relationship, there’s no differentiation between a CHAG or GHS facility.

I: You mentioned CHAG, Church health directorate *(name deleted)*, district director... Aside from them, who else was involved?

R: Yes, the district director visited immediately after the case was reported to the district. He was the first person to come here, which is why he was the one we informed about the likely staff shortage. He assured us he was going to help. After his visit, we had a Zoom meeting with our Medical Director, followed by an in-person visit the next day. Then the diocesan director also came around, followed by the Church institution *(name deleted)* and others.

I: So, I’m assuming that your initial contact with the district director was made through a phone call rather than a formal letter?

R: Yes, just a phone call. As I mentioned, he passes by here every Monday for updates.

I: Aside from all of this, are there any other challenges we haven’t asked about that you experienced while handling the Marburg disease?

R: One challenge I noticed was with sample collection. We had to rely on the district directorate to take samples from the lab. I remember a conversation with the nurse manager, who expressed frustration that some samples were just sitting in the lab, and the directorate wasn’t coming for them. Later, we were told by WHO that Ghana is Marburg-free, so they never came for those samples. But if we were mandated or had access to take the samples ourselves, I know we would have taken a lot more. Sometimes, you have to call repeatedly before the disease control officer comes to pick up the samples. Recently, when we went for our performance review at the district, this issue was discussed thoroughly, and going forward, they promised to improve. I would say it has been somewhat addressed at the performance review meeting.

I: Considering the architectural design of your facility, .......... *(name of facility deleted)*, do you think the positioning of, say, the ICU, isolation unit, or OPD makes your facility more vulnerable to outbreaks? You mentioned that there’s no ICU, but rather an isolation unit.

R: Yes, it’s not a proper ICU—just a room. So, if we have four cases coming in, where will we keep them? Where will they gown and enter, and how will we ensure that no virus is being spread as people leave? There’s no room for gowning. So, they have to go to the emergency unit to gown before entering. Although they’ve improvised ways to ensure the disease doesn’t spread, I don’t think it’s the best solution. We need a well-structured space to ensure proper IPC (Infection Prevention and Control) practices, but for now, it’s improvised.

I: We’ve covered all our questions. Do you have anything more to add?

R: For administrators, I would ask if there are plans to help us build an ICU unit because we are a referral center, not just for the district *(name deleted)*. We also receive cases from Bekwai, New Edubiasi, and other areas. We’ve been discussing this, as we wonder how we would handle it if there were a large number of cases all at once. We would like CHST to please help us. We have land, so we can make land available.

I: Do you have any questions for us?

R: I didn’t really understand the objective of the interview.

I: The objective is multifaceted. For your part, one objective was to examine the psychological impact of quarantine on health workers. We are also looking at how collaboration with other agencies helped control the spread of the Marburg disease, the structures in place, and the emergency response plan for combating Marburg and other disease outbreaks.

R: Ok.

I: Thank you.

DEPUTY CHIEF DISEASE CONTROL OFFICER (DDCO)

| PSEUDONYM | |
| --- | --- |
| PARTICIPANTS | R |
| INTERVIEWERS | I |

*Demographic information was deleted to protect the participants’ identity*

I: Can you describe how the Marburg disease was detected in your district?

R: In the district *(name deleted)*, we have a CHAG (Christian Health Association of Ghana) hospital that serves as a referral point for the district. We also have a health center that functions as a sub-district health center. The person involved was at Takwa initially, and when he came here, he was already sick. He came to the health facility, but they didn’t admit him. They treated the minor illness he presented with and then discharged him. Two days later, he fell sick again. Instead of returning to that facility, he went to ............ Hospital *(name of facility deleted)*, where they detected the issue. He went there for malaria treatment, and while they found malaria parasites, they also observed some strange signs and symptoms. At that time, the medical superintendent wasn’t available, so another medical officer called the medical superintendent, who then contacted my director. My director then called us. That particular day, we rushed to ...........*(name of facility deleted)*, but by the time we arrived, they had already taken samples. However, the sample was not taken specifically to detect Marburg; it was taken for their routine lab work. Later, the doctor called, and we agreed that due to the unusual signs and symptoms, further testing was needed. My director contacted the regional director and the Deputy Director of Public Health (DDPH), and they gave us the go-ahead. You know, with suspected cases of Marburg or Viral Hemorrhagic Fever (VHF), you’re supposed to send samples to Accra within 24 hours. But because the sample was initially taken for routine checks, it was collected on the 26th, and we kept it for three days before sending it to Accra. In Accra, there was also a delay of about two weeks before we received feedback from Noguchi Memorial Institute.

I: Was it the district that took the sample to Noguchi?

R: Yes, I personally left here around 10 in the morning and made the journey. I took two samples with me—one from our district and one from another district, Asante Bekwai District, which is a municipality. Since the CHAG hospital is located here, we included that sample because the person also showed similar unusual signs and symptoms. When we arrived at Noguchi, we submitted two samples: one from Ankaasi and one from here. Initially, the results showed two positive cases. However, after conducting their investigation, Noguchi decided to send the samples to Dakar for further testing. When Noguchi’s results came back positive and Dakar’s came back negative, it created some controversy. People started questioning Noguchi’s findings and favoring Dakar’s results, questioning the reliability of Noguchi’s tests. You understand what I mean, right? It was frustrating because Noguchi was assigned to conduct the investigation and provided positive results. But in the end, people seemed to believe Dakar’s results instead.

I: When the sample was sent to Dakar, did their result come back negative?

R: Yes, when the results came back from Dakar, the sample from the facility *(name deleted)*  was positive, but the sample from the other facility *(name deleted)* came back as negative. They explained that the sample from the other facility *(name deleted)* was insufficient to conduct a thorough investigation.

I: So they did not conduct the investigation at all?

R: Yes, they told us that the sample was not enough. They called and asked me to return to the morgue to take another swab. We have the necessary equipment at the morgue to take swabs from the mouth and nose. After taking the new samples, we sent them back to Dakar. However, this took more than two weeks because the body had been injected with chemicals, which can affect the sample results. Eventually, a pathologist from Komfo Anokye Teaching Hospital came, helped with the swabbing, and we sent it back. Later, the results came back as negative.

I: You mentioned that you had to keep the sample for about two days because it was more like a routine check you were doing. How secure was the sample, given the possibility of people being exposed?

R: In Disease Control, we work closely with lab personnel who are also trained in handling infectious materials. Before any action is taken, we consult with them and the medical officers. The lab has specialized refrigerators for storing samples. The sample was kept securely in one of these fridges, wrapped, and placed in a zip-lock bag, with the name, age, and other relevant information labeled. When transporting it, we used a vaccine carrier with ice packs. From storage to transport, the handling process was secure.

I: You mentioned that when the sample was taken to Dakar, you needed to return and take another swab from the deceased person for retesting. How secure was the morgue, given the potential infectious nature of this disease?

R: When the incident occurred, based on the signs and symptoms the person showed, we were concerned about infection control. The person passed away on the 27th, a day after we took the sample. After I sent the sample to Accra and returned, we met as an emergency preparedness team at the district level. When we encounter a strange disease in the district, our team discusses the issue and decides on immediate actions. My director, the team, and I agreed not to wait for results before taking action. We trained volunteers and the morgue attendants. We explained the situation and the suspicion of a certain disease to the morgue attendants and instructed them on how to handle the body properly. The owner of the morgue, a pathologist from Komfo Anokye Teaching Hospital, also received training. The regional director communicated with him, and he came in to retrain his staff as well.

I: There was a possibility that by the time you received the results, the body might have been released to the family for burial.

R: Yes, because of that, after the person passed away and the body was taken to the morgue, we went to the community to meet with the household and other community members. We took down the names, ages, occupations, and other details of the close contacts, relatives, and friends. We monitored these individuals for 21 days. If they remained symptom-free during this period, they were considered safe. This was not quarantine; we simply explained to them to report any symptoms like headaches so we could investigate further.

I: As part of the process, did you conduct any investigation to determine the virus's origin?

R: Yes, a team conducted a survey in the forest. On their way to Accra, they had some... well, findings. By the time they reached the Eastern Region, they noted the possibility of bats carrying and spreading the virus within 24 hours if infected. They engaged volunteers and encouraged people not to consume bats, monkeys, or similar animals as a precaution.

I: If we extend this to the facilities the first patient visited before arriving at ...........*(name of facility deleted)*, what kind of engagement did you have with those facilities?

R: After the case was identified, we invited the .......... health facility *(name deleted)* to screen the staff members who were present when the patient came in. We collected samples from them and sent them to Noguchi for testing. We also quarantined the staff who had been in close contact with the patient for 21 days.

I: In terms of resources for managing or detecting cases and sharing information within the facility, what kind of support did the District Health Directorate provide?

I: You know, this viral hemorrhagic fever—it's actually fortunate that it happened at a time when we already had the COVID logistics on hand. We were able to use part of those resources to protect the nurses and other staff. We even distributed some sanitizers to community members, and the educational outreach went well.

R: Yes, aside from the face masks and sanitizers, we provided disinfectants to the facility. I was personally there, and the medical doctor *(name deleted)* instructed them to disinfect the area where they admitted the patient. Eventually, they designated a specific room, so whenever someone came in, they’d direct them to that room first. We managed well with the available COVID logistics, but there were financial challenges. For instance, when we traveled to Accra, the fuel costs added up, but they did give us a small amount to cover that. They explained that they needed to request further support from donors.

I: When the disease was first detected, you mentioned a meeting was held to decide on the course of action. Was the decision based on an emergency disaster management or response plan that already exists in the hospital, or is there no such plan in place?

R: At the beginning of the year, the district forms an emergency committee. We meet to discuss and prepare because we can't predict what might happen, so we stay ready. Anytime we receive alerts from the lab or medical officers about a developing situation, we quickly respond. But when an emergency does occur and resources are insufficient, we reach out to the regional authorities or others for assistance.

I: Just to clarify, does the facility have an Infection Control and Prevention (ICP) plan or an emergency prevention and control document? Is there a set document that outlines a step-by-step process to follow during an emergency?

R: Yes, we do have some resources for such situations.

I: How helpful were those resources? I ask because each situation can vary, and every case has its unique aspects.

R: We have an outbreak response guide in Ghana; there’s a manual for reference.

I: So, applying that guide in this case—given that each case can be unique—was it straightforward to follow, or did you need to improvise?

R: Typically, we rely on something called "case definition." We even have laminated posters with case definitions. Some diseases share similar symptoms, like malaria and typhoid, so we base assessments on the symptoms presented. The patient initially arrived at the facility with vomiting, nausea, diarrhea, and later, symptoms like bleeding from the eyes and nose appeared. At first, you may only see symptoms like diarrhea and headache. In a recent case, the medical doctor *(name deleted)* called me, and I quickly went to a nearby CHAG hospital where a patient was vomiting blood for two days before passing on the third day. We collected a sample, sent it to Accra, and despite the Marburg-like symptoms, it tested negative. The symptoms can be similar to Marburg, but testing sometimes yields a different result. This is why having a clear case definition is important—it helps clinicians identify when symptoms seem unusual. In this recent case, the doctor deserved recognition for his diligence.

I: You’re right. Regarding information sharing—following COVID, it feels like things were stabilizing, but now there’s another dangerous virus. With some health workers having had contact with the patient, how was information shared between the directorate, ......... Hospital *(name of facility deleted)*, and the community to prevent further spread?

R: Not every disease with prevalent cases prompts widespread information sharing. Sometimes we withhold or carefully word the information before sharing it. For instance, with Marburg, if you disclose an outbreak in a specific community, certain staff might panic or spread the news too freely. My landlord even asked me about rumors of an outbreak, but I only mentioned COVID to avoid spreading alarm. So, sometimes we keep the information contained.

I: Eventually, how did you communicate the necessary protocols, especially to the patients in the facility? They might notice new protocols—like restricted areas or limitations on ward access. How was this handled within the facility?

R: We adhered to COVID protocols, such as hand washing, face masks, and sanitizing. Instead of specifically announcing a new outbreak, we reinforced COVID safety measures, which people were already familiar with. We placed Veronica buckets for hand washing at the entrance, posted signs for mask-wearing, and communicated that everyone must comply.

I: In your opinion, does the public health system in the District *(name deleted)* have the capacity to handle large outbreaks? Suppose Marburg had spread further—would the district be equipped to manage a widespread situation?

R: It depends on the rate of spread. During COVID, when the outbreak intensified, one of the adjoining cities *(name deleted)* called on our facility *(name deleted)* and other neighbouring districts for assistance. If something similar happens here, we can invite neighbouring districts like that city *(name deleted)* or a close one *(name deleted)* for support. But if it’s within a manageable range, we’re prepared. We have trained nurses and community volunteers who can identify people showing symptoms, and our facility has sufficient staff.

I: Can you describe your relationship with CHAG and the Church institution *(name deleted)* in managing this outbreak?

R: I can’t fully describe the relationship between the directorate and CHAG, but here in .........*(name of district deleted)*, they play a crucial role in resource allocation. They don’t frequently request supplies but instead rely on fair allocations from the store. Their collaboration is strong, and they promptly report any suspicious cases to us. So, the relationship is cordial.

I: Would you say that working with CHAG or other institutions like WHO has been beneficial? Are there any notable experiences, either positive or challenging, in collaborating with these organizations?

R: In public health activities, we often work with community opinion leaders and volunteers. Churches, for example, where people gather, are sites where nurses conduct case searches for diseases like TB. If they observe anyone coughing or displaying unusual symptoms, they refer them to the facility. The trained nurses carry out both passive and active case searches, and they notify medical officers or us if they find anything.

I: What challenges have you faced throughout this process at the facility level?

R: One major challenge is transportation. Our district has only one vehicle, so if the director is using it, we lack another means to travel. Additionally, we don’t have motorbikes here at the District Assembly.

PUBLIC HEALTH NURSE (DPHN)

|  | |
| --- | --- |
| PARTICIPANTS | R |
| INTERVIEWERS | I |

*Demographic information deleted to protect participants*

R: When the Marburg virus was detected, can you explain what the process was like in the detection phase, including sending the samples out? Not so much the clinical details, but rather the managerial and administrative activities that took place.

The client reported to the district hospital on June 26, 2022, with symptoms including fever, diarrhea, and bleeding from the nose and mouth. As a public health unit, led by the then director and the disease control team, we regularly visit the facility and take the clinicians through the case definitions of various diseases, including viral hemorrhagic fevers. When this case occurred, the clinician suspected it might be one of the viral fevers, so the lab personnel took a sample and reported it to the director.

I: So, this means that you had education or information about viral diseases even before the Marburg outbreak?

R: Not exactly formal education, but sensitization for the clinicians so that, even if they see a suspected case of a viral fever, they can identify it, whether it’s an AFC case, measles, or a viral hemorrhagic fever. That way, if they come into contact with it, they can suspect the cause and take the necessary action. So, they took the sample and informed the director, who then notified the regional authorities about the suspected case. We did not initially suspect that it was Marburg; rather, we suspected it could be one of the viral fevers. The sample was sent to Noguchi, and when the results came back, it was confirmed positive for Marburg. The deceased was then transferred to a morgue in another district, as our district does not have one, and the family was advised not to handle the body because we suspected a viral disease.

I: So, for the first case, after the person passed away, did you take the body away from the family? What advice was given to the family for clarification? Were they advised to stay away from the body?

R: Yes, that advice was given days before. However, the client was Muslim, and the family retrieved the body before we received the confirmation that it was Marburg disease.

I: So how did you follow up with the advice to assist them afterwards?

R: After the confirmation, we contacted the relatives to inform them of the outcome and the need to screen all contacts who came into contact with the deceased. Samples were taken from them for laboratory investigation to check if they had also been infected.

I: What steps were taken at the facility?

R: Those at the facility who had been in contact with the deceased were also quarantined for a specified period.

I: When the staff came into contact with the deceased, did they show any symptoms of infection?

R: No, they did not show any symptoms of the infection.

I: It’s our understanding that the patient had been to ....... Hospital *(name deleted)*  before, and after a couple of days, he went to .............*(name of facility deleted)*. Were the people at ........... *(name deleted)* also quarantined?

R: No, they were not.

I: Is there any specific reason why they were not quarantined?

R: The patient went to .........*(name of town deleted)* at night and was seen by the Physician Assistant (PA) and one nurse. The client had tested positive for malaria, so he was treated for malaria and then went back home.

I: Besides quarantining and contact tracing measures, were any other preventative measures implemented to avoid further infection?

R: Yes, the morgue where the client’s body was sent was contacted to disinfect the area where the client was deposited. They were informed of the disease that the client had.

I: What role did your department play in this process?

R: Since this case was at the hospital, their management unit was primarily responsible, in coordination with the district disease control officer. My role was to sensitize the nurses on how to protect themselves, what they were expected to watch for in cases of Marburg, and to remind them to refer any suspected cases to the facility if they encounter them in the community. Once a case was confirmed, the possibility of others contracting the virus increased, so we provided guidance.

I: Can you describe the type of sensitization you undertook, the objectives of each, and how the staff were trained? Was it through a webinar, or was it done in person?

R: We have four sub-districts, and the patient came from ..........*(name of town deleted)*, so we started the training there, followed by .............*(name of facility deleted)*. Staff were trained on the case definition, signs, and symptoms, as well as precautions they needed to take. At that time, COVID-19 was also a concern, so we reminded them to wear masks, wash hands frequently, and use hand sanitizer.

I: You were about to mention another point.

R: Yes, we also included the Community Health Nurses (CHNs). We trained them on the disease, its causes, and how it spreads, and they were employed to go house-to-house to talk about the disease, its causes, signs, and symptoms, so that if they observed any suspected case, they could quickly refer it to the facility.

I: In terms of your internal processes with patients at the hospital during that period, how did you engage them with information on symptoms to watch for?

R: The client was isolated in a separate room, but I cannot speak much about engagement with other patients.

I: What role did the district play in supporting patients and families who were infected?

R: My director and the disease control officer mainly handled this. They visited homes and called family members on the phone to check on them and ask if they were experiencing symptoms like fever. At one point, relatives didn’t want us coming to their homes in our vehicles because they feared stigma. To address this, even when national health officials visited, they used taxis so that the community wouldn’t notice. Family members were mostly contacted by phone and in person to reassure them.

I: So, there wasn’t any material support provided?

R: No, nothing like that.

I: In terms of the ..............*(name study facility deleted)* and .......... facilities *(name of another facility deleted)*, what kind of supplies did they receive from the directorate?

R: They received posters on the disease, hand sanitizers, chlorine solutions, face masks, gloves, and other materials for infection prevention and control.

I: What considerations were made regarding the type of supplies given to ..........*(name of study facility deleted)*, .........*(name of another facility deleted)*, or any other facility?

R: Knowing that Marburg disease is highly infectious and with COVID-19 also present, there was a need for plenty of disinfectants, like chlorine and hand sanitizers, to protect staff. Fortunately, the district had sufficient supplies in stock, so we distributed them to protect staff.

I: In terms of the community, what was done?

R: For the community, we focused on awareness creation. We went to the Community Information Center (CIT) and used posters since we don’t have a radio station.

I: Does the district directorate have a disaster management and response policy in case of a disease outbreak?

R: Yes, we have a committee, which includes District Chief Executives (DCEs) and other stakeholders, that convenes in response to outbreaks.

I: Is there any document that prescribes the steps to follow at the district level?

R: No, but recently, the disease control officer and the health promotion officer attended a review meeting at the national level about Marburg, and a policy was discussed. It outlines expected steps to follow in case of an outbreak.

I: Could you provide us with the name of that policy document, or could you text it to us later?

R: Yes, I will ask them and get back to you.

I: We discussed how you communicated information to your staff, but what about people within the facility? Grace will proceed from here.

Grace (I): In terms of public health capacity, we understand that ............. *(name of facility deleted)* does not have a public health person, so were you the one responsible?

R: There is a public health nurse and a health promotion officer at .........*(name of facility deleted)*. What they lack is a disease control officer, but the public health unit, including a PHN, is present.

I: How is the working relationship between the directorate and the facilities, especially the district hospital in ..................*(name of facility deleted)*?

R: .................*(name of facility deleted)* is our district hospital, so we involve them in everything unless instructions from the region specify otherwise. We collaborate closely, share consumables, commodities, and funds, include them in performance reviews, and monitor them. Recently, our new director coordinated with the medical doctors *(name deleted)* to lead and train our midwives in obstetric emergencies. We have a strong working relationship.

I: Apart from ....................*(name of facility deleted)*, are there other CHAG facilities in your district?

R: No.

I: What challenges do you face with disease surveillance and emergency response in the facilities as a district directorate?

R: With active surveillance, we are supposed to get some … and go into the communities, especially the hard-to-reach communities where people cannot come to the facility for passive surveillance. The staff, like the CHNs and field technicians, are supposed to do active surveillance, but this requires funding. Unfortunately, the funds aren’t always available for active surveillance, so we mostly rely on passive surveillance. This means that clients come to the facility, and if clinicians suspect a case, we take it from there. Clinicians sometimes need reminders about case definitions and staying vigilant in identifying suspected cases, as they may forget without proactive encouragement. Periodically, we conduct sensitization sessions to keep our clinicians alert. For instance, in their performance appraisal, facilities under the GHS (Ghana Health Service), especially facility heads who are clinicians, are expected to report a number of AFPs and other cases. This appraisal process helps prompt them to look out for these diseases.

I: You mentioned periodic sensitization. How often does this happen? Is it quarterly, monthly, or …?

R: It’s not on a strict schedule. We usually address it whenever we go for monitoring visits to the facilities. When we meet with clinicians and lab staff, we talk to them about staying vigilant for suspected cases. So, it’s not structured like a regular training program, but during any monitoring visit, whether it’s for HIV or another area, we remind them of the importance of being alert for suspected cases.

I: So, there’s no specific schedule; you just address it as needed.

R: Yes, exactly. For ZB (Zoonotic and Biohazard) funding, funds do come to the directorate to support disease control, and the director often organizes sessions to sensitize staff and clinicians on these matters. We have coordinators who come to the directorates, and the disease control team and the director guide them on what needs to be done in terms of ZB management during that period.

I: Considering diseases like Marburg and even COVID, which isn't so far removed, how do you manage information sharing between the district and the facilities under you?

R: We use a WhatsApp platform since some facilities are quite distant from the directorate. We share a lot of information through these WhatsApp platforms.

I: Who are the members on these platforms? Are they administrators or medical superintendents?

R: They’re all on the platform: administrators, nurse managers, everyone. We have specific platforms, such as one for staff and one for AEFI (Adverse Events Following Immunization), so we have various platforms for different purposes.

I: Do you have a single platform where the admin, nurse manager, med sups, and everyone are all together, or are they on separate platforms?

R: Yes, we have one platform that includes everyone, for all staff.

I: And that’s where you start disseminating the information?

R: Yes, we post information there first, especially since letters can sometimes be delayed in reaching them. By posting it on the platform, they get the information before the hard copy arrives.

I: Do you have anything to add, maybe something we haven’t asked, or do you have any questions for us?

R: Okay. One other case came up from Bekwai. Initially, Noguchi Institute said it was positive, but later they mentioned that the sample brought to them wasn’t sufficient, so they asked us to provide another sample. By that time, the deceased had already been sent to a morgue and frozen, so we couldn’t retrieve another sample. As a result, they later reported it as negative, which means that in the end, we only had one confirmed positive Marburg case from the Ashanti Region.

I: Thank you for sharing that.

R: Another point to note is that hunters, farmers, and people involved in bush meat trade were also engaged in risk communication. We gathered them from all the sub-districts to one location and explained the Marburg disease to them. We educated them on how to handle meat safely. We advised them that if a hunter goes into the bush and finds a dead animal, they shouldn’t pick it up and sell it. The environmental and veterinary officers also participated in this training.

I: Thank you very much.

HUMAN RESOURCE MANAGER (HRM)

|  | |
| --- | --- |
| PARTICIPANTS | R |
| INTERVIEWERS | I |

I: When the virus was detected in the facility, as the HR, based on your observations, how was the information communicated to you, and what adjustments were made immediately after the virus was detected?

R: When the case first came, it was entirely new to us. It wasn’t until the results of the samples came out that we realized the severity of the situation. Management quickly conducted an emergency meeting to discuss the findings, the position of management, and the steps they would take to handle the situation effectively. The first action was to ask everyone who had come into contact with the cases, primarily the prescribers and nurses in direct contact, to isolate themselves. This was done to prevent any possible spread of the virus, in case there was one. That was the initial step taken by management. At that point, my role as HR came into play, especially since these individuals were isolating. With their absence, we needed additional hands to ensure the smooth running of operations. I believe there were around three prescribers and a number of nurses from the emergency unit involved. So, we sent out letters to the district, region, and all relevant bodies to request temporary staff to cover for those in quarantine. This was the primary response management took, alongside some education on how to handle future cases.

I: As the HR, I assume you’d be particularly concerned with the welfare of your staff. Could you tell us about the protective equipment provided to those who were still on duty, as well as the new staff brought in to manage the situation?

R: Honestly, management did a commendable job in making sure that those who were isolating were well taken care of. Since our facility was the first to record these initial cases, they provided all the essentials needed for staff in quarantine.

I: What specific equipment and support were provided to them?

R: They were provided with PPEs, and arrangements were made to ensure that their isolation was well-supported. Food, shelter, and all basic needs were made available at their isolation locations. Everything was provided to prevent them from having to leave or take things to their own homes until the isolation period was complete and final results confirmed they were negative.

I: What about the well-being of the staff who were quarantined? How were you involved in ensuring their welfare was managed?

R: There was a committee set up to oversee their welfare specifically. My role mainly focused on coordinating letters and requests to secure staff replacements during the period of their quarantine.

I: So, you were responsible for bringing in the replacement staff?

R: Yes, exactly, in their absence.

I: And in terms of their welfare during quarantine, was that handled by the committee?

R: Yes, everything they needed was provided. I was also aware of what they received since supplies, like food and water, were stored in an office near mine. The drivers would collect the items and distribute them to each individual in quarantine, so I was fully aware of the provisions they were getting.

I: Did you receive any complaints from the staff regarding the measures that were put in place?

R: Actually, it was a mix of complaints and recommendations. Some of them even suggested extending the quarantine period if such provisions continued to be made available.

I: What about the new staff you brought in to cover for those in quarantine? Were there any complaints from them about the resources they needed or about any gaps in managing the situation?

R: Interestingly, a few of the new staff members were among those who went into quarantine. One of them actually came to my office, unaware of the quarantine status. I needed him, and I didn’t realize he was supposed to be in quarantine. He later told us he was part of the first group meant to isolate. I asked him what he was doing at the office since the whole unit was quarantined. Since the new staff hadn’t seen how intense COVID-19 was in other facilities, they seemed to view this as a manageable situation and didn’t seem panicked or worried. It was only two cases, and it was well-managed, so they didn’t really have any complaints.

I: In terms of training, you mentioned a meeting was held where staff were informed about the situation. Could you elaborate?

R: An emergency meeting was called by management, mainly involving management-level staff to avoid crowding. The information was then cascaded down to the rest of the staff through unit representatives, like the nurse managers and other departmental heads. This helped us communicate the situation clearly to everyone without gathering all the staff in one place.

I: Aside from that, was there any specialized training provided to the staff? Marburg is a new virus in the region, unlike COVID-19 or Ebola. Was there any training planned specifically for Marburg?

R: Yes, the nurse manager took the lead in training the nurses. She invited the disease control team and the medical director also contributed to the training. We made sure that everyone received the necessary guidance while keeping gatherings small to minimize any risks.

I: From your perspective, even after bringing in additional staff, do you think the facility had enough human resources, both in numbers and skill levels, to handle the outbreak effectively?

R: The facility truly did its best under the circumstances. Although the number of available personnel wasn’t particularly high, we worked as a team and supported one another to reach a common goal. Management put in great effort, and everyone worked together to manage the situation effectively.

I: So, in summary, would you say the resources weren’t entirely sufficient to deal with the situation?

R: Yes, and I remember reading some of the news reports that made it seem like the situation was more intense than it was. But in reality, it was well-controlled and quickly returned to normal.

I: Concerning the welfare of the staff, considering that Marburg is a highly fatal disease, even more so than COVID-19, was there a support system like a counseling desk available for staff who might have felt anxious or traumatized?

R: We have a health team that works on such cases daily, and we also have a chaplain available on-site who offers support. If the health team finds a case they feel they cannot manage, they refer it to the chaplain, who provides further assistance. This was done regularly.

I: Did you receive any reports suggesting that some staff were experiencing psychological distress?

R: No, not really. From that time until now, I haven’t received any such reports.

I: Do you have a morgue at the facility?

R: No, we don’t.

I: What about an ICU?

R: Yes, I’m actually located in the newly constructed ICU right now.

I: Was it built after the Marburg outbreak?

R: No, it was a long-term project that had been ongoing. Our facility isn’t very large, but we do have a spacious compound. The ICU is relatively small, but we have equipped it with the necessary resources.

I: Considering the architectural design of your facility, its location, and the proximity of wards to staff and clients, do you think there’s anything about the setup that might predispose your facility to future outbreaks?

R: Not really.

I: While managing the outbreak, you had to handle quarantine for some staff, coordinate replacements, and follow clinical protocols. How did all of this impact your personal life, especially how you relate to people and your perspective on work?

R: This experience affected me positively. It has motivated me to focus more on certain aspects of my work that I may have previously overlooked. For example, as HR, I learned that motivating staff isn’t just about monetary rewards; there are other ways to encourage them. Seeing the staff happy and engaged due to these new approaches has been a rewarding experience.

I: Was there anything during this period that made you feel particularly anxious, and if so, how did you cope with it?

R: I don’t recall feeling any specific anxiety. For us, certain situations like these have become part of our routine.

I: Do you live with your family?

R: Yes, I do.

I: When this happened, did it affect your relationship with them, knowing you might be bringing a virus home?

R: Fortunately, my wife is also a health worker, so we’re used to managing these kinds of situations. We also educated our kids on how to stay safe, so it wasn’t much of an issue.

I: We’re almost done. Just a few questions about the facility's collaborations. How would you describe the relationship between your facility and the Ghana Health Service in managing outbreaks?

R: Since our facility is within the district, we have an ongoing collaboration with the district health authorities. For instance, during the outbreak, the District Health Director was frequently involved, working closely with our medical director to handle everything together.

I: Could you tell us what kind of assistance you received from the district directorate beyond the support for staff?

R: We received quite a bit of assistance. Since COVID, our district’s disease control unit has worked closely with us, especially in health education and other joint efforts. We collaborate as a unified team to ensure the best outcomes.

I: Did you receive any PPEs from the district?

R: I’m not entirely sure about that. All I know is that the necessary supplies were often obtained from our stores.

I: Did the district assist with contact tracing efforts?

R: Yes, as I mentioned, contact tracing was a joint effort between our facility and the district. Collaboration is the norm, both in previous outbreaks and in this one.

I: Was there any collaboration with CHAG during this outbreak?

R: Yes, the CHAG director came down personally as soon as he heard that one of their facilities had recorded a Marburg case. They were directly involved and aware of everything. We even had a letter requesting additional staff to help in the absence of those in quarantine.

I: Are you aware of the specific assistance that CHAG provided during that period?

R: Most of that information would go to the diocesan secretary. When the CHAG director came, he coordinated with the diocesan secretary to manage any supplies or assistance needed.

I: Aside from CHAG and the Ghana Health Service, did any other agency, public or private, offer assistance during the outbreak?

R: No, not that I’m aware of.

I: Were you involved in the process of sending samples to Noguchi and receiving results? How did that process go?

R: Yes, and that’s what prompted management to call an emergency meeting to inform everyone about the results and the steps we needed to take in collaboration with the District Health Director and Regional Health Director.

I: So, the process of sending samples to Noguchi and getting the results back was smooth?

R: Yes, very smooth. The quick turnaround allowed us to act promptly, which was crucial.

I: We heard that the first case was referred to your facility, and it was only then that Marburg was detected. How did your facility work with the previous facility in investigating further and containing the spread?

R: Our medical director didn’t hesitate. She personally followed up to ensure that the right steps were taken, including tracing the contacts of the deceased and those involved with the family.

I: In contacting agencies during the Marburg outbreak, were there times you used informal approaches, like making a phone call, to get assistance quickly?

R: Yes, the only informal approach we used was phone calls. However, I consider phone calls a necessary form of communication, especially for urgent matters.

I: Thank you very much, Mr. Agyeiman. We’ve covered all our questions. Is there anything else you’d like to add about your role in managing the virus?

R: No, I think you’ve asked all the important questions.

I: Do you have any questions for us?

R: No.

I: Thank you very, very much for your time, Mr. Agyemang. We appreciate you taking the time away from your office.

R: Haha, it’s normal.
